# SeekFusion - A Clinically Validated Fusion Transcript Detection Pipeline for PCR-Based Next-Generation Sequencing of RNA

**DOI:** 10.3389/fgene.2021.739054

**Published:** 2021-10-22

**Authors:** Jagadheshwar Balan, Garrett Jenkinson, Asha Nair, Neiladri Saha, Tejaswi Koganti, Jesse Voss, Christopher Zysk, Emily G. Barr Fritcher, Christian A. Ross, Caterina Giannini, Aditya Raghunathan, Benjamin R. Kipp, Robert Jenkins, Cris Ida, Kevin C. Halling, Patrick R. Blackburn, Surendra Dasari, Gavin R. Oliver, Eric W. Klee

**Affiliations:** ^1^ Quantitative Health Sciences, Mayo Clinic, Rochester, MN, United States; ^2^ Division of Laboratory Genetics and Genomics, Mayo Clinic, Rochester, MN, United States; ^3^ Applied Genomics Division, Perkin Elmer, Waltham, MA, United States; ^4^ Division of Anatomic Pathology, Mayo Clinic, Rochester, MN, United States; ^5^ Information Technology, Mayo Clinic, Rochester, MN, United States; ^6^ Department of Pathology, St. Jude Children’s Research Hospital, Memphis, TN, United States

**Keywords:** gene fusion, RNA, bioinformatics, UMI consensus, QiaSeq, sarcoma, neuro-oncolgy, neuro-oncological disease

## Abstract

Detecting gene fusions involving driver oncogenes is pivotal in clinical diagnosis and treatment of cancer patients. Recent developments in next-generation sequencing (NGS) technologies have enabled improved assays for bioinformatics-based gene fusions detection. In clinical applications, where a small number of fusions are clinically actionable, targeted polymerase chain reaction (PCR)-based NGS chemistries, such as the QIAseq RNAscan assay, aim to improve accuracy compared to standard RNA sequencing. Existing informatics methods for gene fusion detection in NGS-based RNA sequencing assays traditionally use a transcriptome-based spliced alignment approach or a *de-novo* assembly approach. Transcriptome-based spliced alignment methods face challenges with short read mapping yielding low quality alignments. *De-novo* assembly-based methods yield longer contigs from short reads that can be more sensitive for genomic rearrangements, but face performance and scalability challenges. Consequently, there exists a need for a method to efficiently and accurately detect fusions in targeted PCR-based NGS chemistries. We describe SeekFusion, a highly accurate and computationally efficient pipeline enabling identification of gene fusions from PCR-based NGS chemistries. Utilizing biological samples processed with the QIAseq RNAscan assay and in-silico simulated data we demonstrate that SeekFusion gene fusion detection accuracy outperforms popular existing methods such as STAR-Fusion, TOPHAT-Fusion and JAFFA-hybrid. We also present results from 4,484 patient samples tested for neurological tumors and sarcoma, encompassing details on some novel fusions identified.

## Introduction

Gene fusions are potentially pathogenic events that result from genomic structural rearrangements including inversions, translocations, and interstitial deletions. Gene fusions are frequently observed in cancer, and while their prevalence varies by tumor type they have been estimated to account for 20% of all cancer morbidity (Mitelman et al., 2007). Identification of gene fusions in a tumor can help guide therapeutic decision-making since fused protein products can represent targets of small molecule inhibitors or other novel treatments. Examples include the treatment of recurrent PTPRZ1-MET fusion-positive glioblastoma using the MET kinase inhibitor crizotinib, KIAA1549-BRAF fusion driven pediatric pilocytic astrocytoma treatment using the MEK inhibitor trametinib, and ALK-EML4 fusion-positive lung cancer using tyrosine kinase inhibitor lorlatinib (Bender et al., 2016; Jain et al., 2017; Shaw et al., 2019).

RNA sequencing (RNA-Seq) assays for gene fusion detection provide improvements in throughput, sensitivity and specificity over traditional DNA and protein-based approaches like fluorescence *in situ* hybridization (FISH) and immunohistochemistry (IHC) (Wang et al., 2009; Abel et al., 2014; Moskalev et al., 2014; Pekar-Zlotin et al., 2015; Haynes et al., 2019). RNA-based strategies to detect gene fusions use transcriptome-wide sequencing with ribosomal RNA-depleted, fractionated messenger RNA or target specific genes of interest using polymerase chain reaction (PCR) based amplicon RNA-Seq, or bait hybridization (capture and ligation) using assays such as QIAGEN’s QIAseq RNAscan panel or Illumina’s SureSelect RNA capture (Wang et al., 2009; Blomquist et al., 2013; Drilon et al., 2015). The PCR-based amplicon RNA-Seq methods offer inexpensive and highly accurate sequencing of fusion transcripts, simultaneously assessing dozens to thousands of targets (Blomquist et al., 2013) and enabling detection of lowly expressed fusions at a high sequencing depth. While PCR-based approaches have traditionally been limited due to stochastic errors that propagate to all PCR cycles (Thilly, 1993), the addition of unique molecular indexes (UMI) in ligation adapters has recently been used to alleviate the impact of such errors (Kivioja et al., 2012), and can improve the sensitivity of gene fusion detection assays. QIAseq NGS assay panels have been demonstrated to provide robust correlation with RT-PCR and low PCR-bias (Wong et al., 2019), and so was the chemistry used in this study (see *Methods*, *Library Preparation and Sequencing* section).

Several bioinformatics tools are available for detecting gene fusions that use wide variety of approaches. Brian et al. and Trung et al. have each outlined benchmarking of 15 gene fusion identification tools in their studies and detailed the methods and performance (Vu et al., 2018; Haas et al., 2019). One common approach uses transcriptome-based spliced alignment. This method is reliant on the accurate mapping of short reads to the transcriptome, which can be challenging due to genome repetitiveness, sequence homology, incomplete transcriptome annotations, and novel patient sequences not well-represented by the reference genome. Matteo et al. elaborate on limitations of several gene fusion identification tools using short read alignment technologies and report key factors such as read length, quality scores and number of reads supporting each fusion call (Carrara et al., 2013a). *De-novo* assembly-based approaches yield longer contigs from short reads and address some of the limitations of short-read alignment but are computationally intensive and often not scalable to large scale clinical testing (Carrara et al., 2013b; Davidson et al., 2015; Haas et al., 2019). Hybrid approaches such as the method implemented in JAFFA (Davidson et al., 2015) combine the approaches described above, but so far, no hybrid method achieves a balance of scalability and accuracy.

Herein we describe SeekFusion (https://hub.docker.com/repository/docker/jagadhesh89/seekfusion), a time-efficient pipeline that leverages *de-novo* assembly and alignment based approaches to accurately identify gene fusions utilizing PCR-UMI-based amplicon RNA-Seq. SeekFusion performs rapid alignment to gene sequences, then groups and filters aligned reads for *de-novo* assembly. Assembled contigs are realigned to a reference genome and fusion genes are identified, annotated and reported in a VCF format. Using verified clinical cases and synthetic controls, we demonstrate that SeekFusion outperforms existing pipelines by balancing high analytical sensitivity and specificity with computational efficiency. The algorithm is written using Python and bash and the functions are wrapped in WDL and processed using Cromwell (Voss, 2017) for ease of deployment by end-users in their own compute environments.

## Methods

### Methods Overview

**FIGURE 1:**
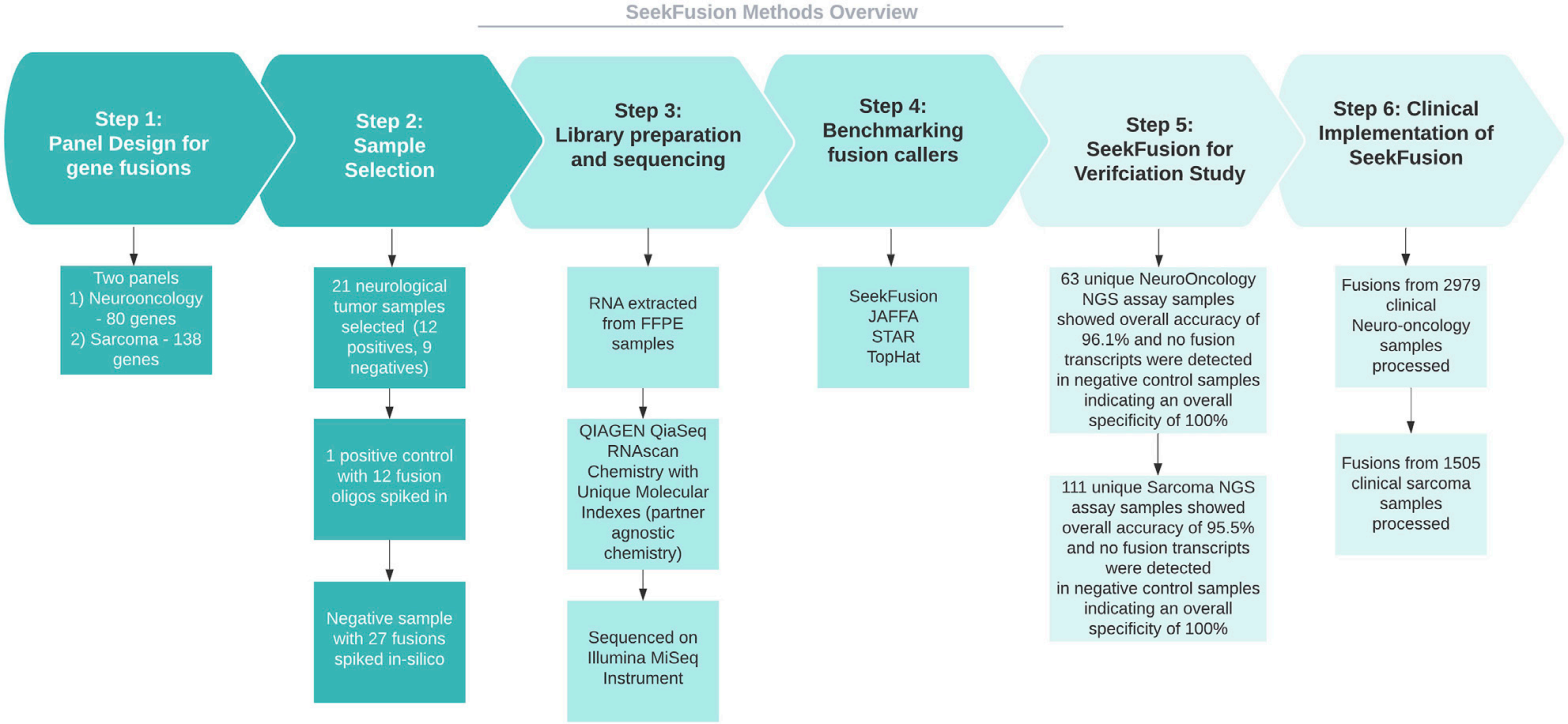
Methods overview. The study involves developing Neuorlogical oncology and Sarcoma clinical NGS assays aimed at targeting gene fusions. Towards development of the assay, few positive samples, negative samples and in-silico samples were selected, sequenced and analyzed across multiple fusion callers. The fusion calls were examined to identify the winning method, and the winning method was SeekFusion, which was internally developed and optimized for the assay. The method was then used towards verification of the assay for NYS approvals and was deployed clinically for gene fusion identification.

The study (Figure 1) involves developing a bioinformatics method for neurological cancer and sarcoma cancer clinical NGS assays aimed at targeting gene fusions. Towards development of the assay, few positive samples, negative samples and in-silico samples were selected, sequenced and analyzed across multiple fusion callers. The fusion callers were benchmarked carefully, results were summarized and compared to orthogonal assay results to identify the winning method. SeekFusion, an internally developed pipeline is highly optimized and accurate compared to the other methods that included STAR-Fusion, JAFFA and Tophat-Fusion. SeekFusion was then used towards verification of the developed assay, the assays were then implemented clinically for gene fusion identification post New York State (NYS) approval for the laboratory developed tests.

### Panel Design for Gene Fusion Detection in Neurological Cancers and Sarcomas

A neurological oncology panel was designed to target fusions in 80 genes (Supplementary Table S2) utilizing the Qiagen QIAseq RNAscan Custom Panel (Blessing et al., 2019). Targeted rearrangements were selected based on association with a variety of adult and pediatric central nervous system (CNS) tumors and potential utility in the differential diagnosis of these tumors or in the differentiation of molecularly defined tumor subtypes (e.g., ependymoma RELA fusion-positive) (Kim et al., 2017). A sarcoma assay was designed to diagnose specific soft tissue and bone tumors (sarcoma) based on the observed gene fusion in 138 genes (Supplementary Table S3). Targeted rearrangements were selected based on associations with a variety of sarcoma types such as rhabdomyosarcoma, synovial sarcoma, and Ewing’s sarcoma. Both neurological oncology and sarcoma assays’ chemistry is designed to target a list of genes known to be involved in rearrangements using gene specific primers but uses a universal primer on the partner gene, making it a partner-agnostic chemistry capable of novel gene fusion identification. Specimen requirements and types for the assays have been detailed in Supplementary Methods section.

### Sample Selection for Benchmarking

Twenty-one neurological tumor samples were obtained following IRB-approved protocols. Of the 21 samples, 12 were fusion positive and 9 were fusion negative (Table 1). In addition to these clinical samples of known gene fusion status, we included a positive control, created from fusion-negative samples to which 13 gene fusion oligonucleotides were spiked in. Finally, a normal control sample was sequenced, and 27 unique fusions were added in-silico as positive controls. In total, benchmarking was performed using 52 known positive gene fusions. All samples were processed using the 80-gene QIAseq RNAscan neurological oncology NGS panel.

**TABLE 1:** List of samples for benchmarking.

Sample ID	Tumor type	Expected fusion	Fusion positive/negative	Confirmed method
pos_S1	Pilocytic astrocytoma (WHO Grade I)	*KIAA1549-BRAF*	positive	RT-PCR with Sanger sequencing
pos_S2	High grade astrocytoma consistent with glioblastoma small cell type, IDH-wildtype	*EGFR-SEPT14*	positive	RT-PCR with Sanger sequencing
pos_S3	Granular cell astrocytoma	*EGFR-EGFR (EGFRvIII)*	positive	RT-PCR with Sanger sequencing
pos_S4	Anaplastic astrocytoma, IDH-mutant	*EWSR1-FLI1*	positive	RT-PCR
pos_S5	Pilocytic astrocytoma	*FAM131B-BRAF*	positive	RT-PCR with Sanger sequencing
pos_S6	Glioblastoma, IDH-wildtype	*FGFR3-TACC3*	positive	RT-PCR with Sanger sequencing
pos_S7	Glioblastoma, IDH-mutant	*MN1-MOB3B*	positive	RT-PCR with Sanger sequencing
pos_S8	Pilocytic astrocytoma	*SRGAP3-RAF1*	positive	CMA
pos_S9	Anaplastic ependymoma (WHO grade III)	*C11ORF95-RELA*	positive	RT-PCR with Sanger sequencing
pos_S10	Pilocytic astrocytoma	*PDE4B-NTRK2*	positive	RT-PCR with Sanger sequencing
pos_S11	Pleomorphic xanthoastrocytoma	*QKI-RAF1*	positive	RT-PCR with Sanger sequencing
pos_S12	Solitary fibrous tumor	*NAB2-STAT6*	positive	RT-PCR with Sanger sequencing
pos_S13	Negative sample spiked in with fusion oligos	*SRGAP3-RAF1*	positive	Specific synthetic oligonucleotide design
		*KIAA1549-BRAF*		
		*MYB-QKI*		
		*FAM131B-BRAF*		
		*PVT1-MYC*		
		*FGFR1-TACC1*		
		*DDX31-GFI1B*		
		*SLC44A1-PRKCA*		
		*MYB-ESR1*		
		*PTPRZ1-MET*		
		*TPM3-NTRK1*		
		*MYB-PCDHGA1*		
		*FGFR1-FGFR1 (E9-E19)*		
pos_S14	Negative cell line with insilico spiked in fusions	*FGFR3-FAM184B*	positive	Insilico spike in
		*MET-ST7*		
		*NTRK1-TPR*		
		*ZSCAN21-MET*		
		*ATG7-RAF1*		
		*MET-PTPRZ1*		
		*ETV6-NTRK3*		
		*PACRG-QKI*		
		*KIAA1549-BRAF*		
		*TFG-MET*		
		*FN1-FGFR1*		
		*NTRK2-VCL*		
		*FGFR3-TACC3*		
		*KIAA1549-BRAF*		
		*QKI-RAF1*		
		*QKI-NTRK2*		
		*NAB2-STAT6*		
		*EGFR-EGFR*		
		*KIF21B-NTRK1*		
		*YAP1-FAM118B*		
		*C11orf95-RELA*		
		*PDE4B-NTRK1*		
		*EWSR1-FLI1*		
		*FAM131B-BRAF*		
		*FGFR1-TACC1*		
		*MYB-QKI*		
		*SRGAP3-RAF1*		
neg_S1	CNS negative	None	negative	CNS negative
neg_S2	CNS negative	None	negative	CNS negative
neg_S3	CNS negative	None	negative	CNS negative
neg_S4	CNS negative	None	negative	CNS negative
neg_S5	CNS negative	None	negative	CNS negative
neg_S6	CNS negative	None	negative	CNS negative
neg_S7	CNS negative	None	negative	CNS negative
neg_S8	CNS negative	None	negative	CNS negative
neg_S9	CNS negative	None	negative	CNS negative

### Orthogonal Validation

Gene fusions detected while benchmarking was confirmed using OncoScan FFPE Assay Kit (Thermo Fisher Scientific, Waltham, MA) chromosomal microarray (CMA) and reverse transcription polymerase chain reaction (RT-PCR) with or without subsequent direct Sanger sequencing as previously described (Gliem and Aypar, 2017).

### Library Preparation and Sequencing

RNA was extracted from macrodissected, unstained slides using the QIAamp miRNeasy FFPE kit and nucleic acid was quantitated with the NanoDrop 2.0 system (Blessing et al., 2019). Library preparation was performed with the QIAseq RNAscan chemistry using 200 ng of RNA per manufacturer’s protocol recommendations. Final libraries were quantitated using a Qubit fluorometer, with 8 equimolar samples pooled and sequenced on an Illumina MiSeq instrument. Raw sequencing data was de-multiplexed into FASTQ files and processed through the SeekFusion pipeline.

### SeekFusion Fusion Detection Workflow

**FIGURE 2:**
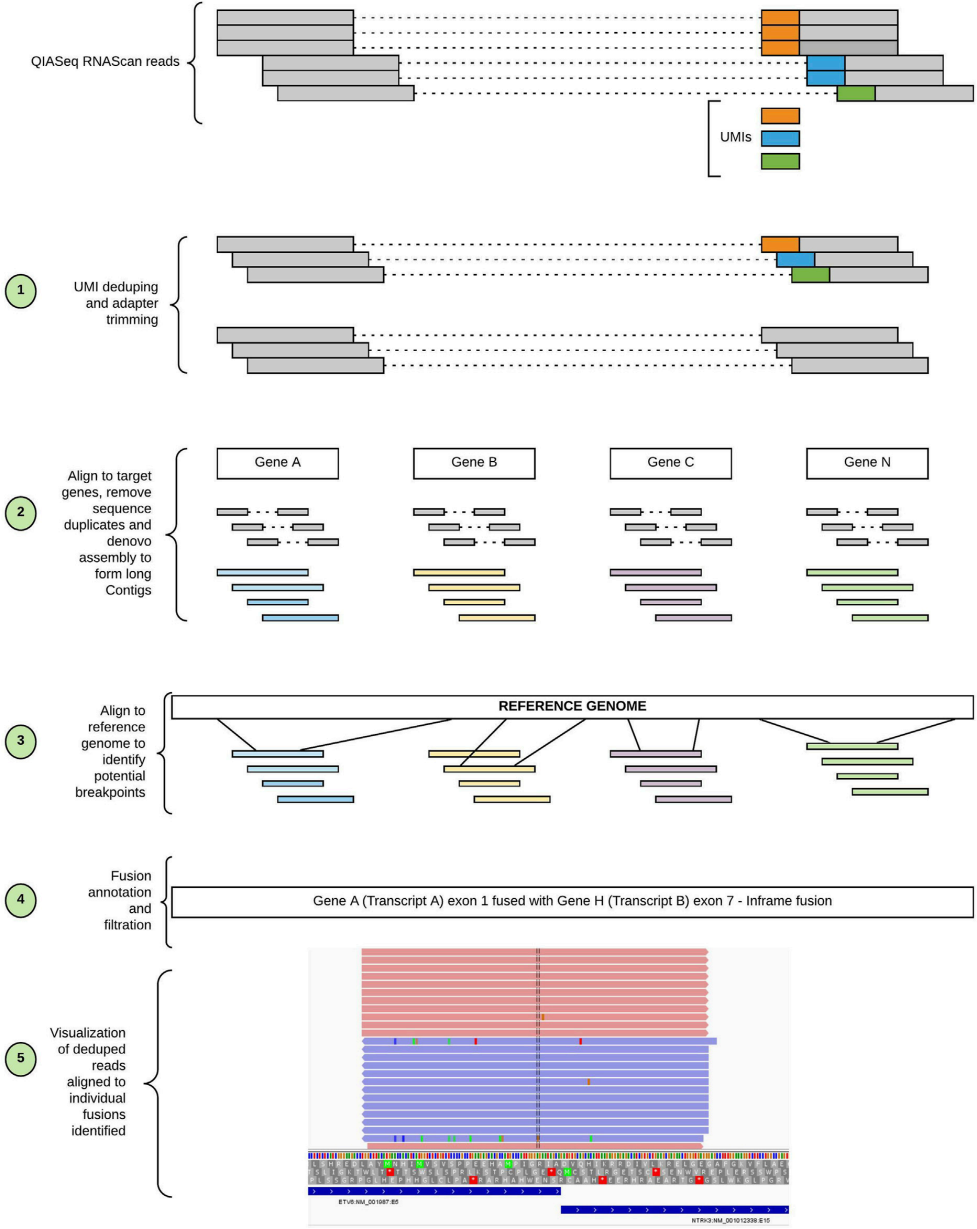
Workflow of SeekFusion pipeline. The pipeline begins with deduping the reads utilizing the UMIs; the deduped reads are then aligned to target genes. The aligned reads are pooled based on the genes and for each gene-based read pool a *de-novo* assembly is performed to construct longer contigs. The contigs are aligned to the reference genome to identify potential breakpoints. The potential breakpoints are annotated, and reads are aligned to the breakpoints to visualize the fusions.

SeekFusion was designed in Cromwell/WDL to enable standalone or server mode workflow execution. Server mode performs tasks in parallel for every gene targeted (Figure 2). The pipeline is configurable for each module, and parameters can be fine-tuned through the profile settings file for the pipeline. Details of the individual steps comprising the pipeline are described in the following sections.

### UMI Based PCR Duplicate Removal (UMI Consensus)

PCR duplication is a byproduct of amplification steps in sequencing protocols that produces multiple copies of individual sequence fragments and artificially inflates their abundance. The QIAseq RNAscan chemistry utilizes UMIs that are random nucleotide sequences incorporated into sequence fragments prior to amplification to enable the identification of PCR duplicate reads originating from the same sequence fragment. Barring technical and sequencing errors, each of these duplicated reads will share identical nucleotide sequence. In reality, however, sequencing errors introduce differences in duplicate reads. The “deduping” module accounts for these errors and attempts to identify the PCR duplicates irrespective of sequencing error. It then builds a single consensus read from identified PCR duplicates and produces an updated Phred quality score for each base that accurately represents the summary evidence when estimating the probability of an erroneous base call. This approach produces higher confidence base calls that facilitate accuracy of downstream processing and improves the ability to discern true calls.

The deduping algorithm assumes a prior probability for the nucleotides at a particular position and uses Bayes theorem to update the probability of the base in a particular position of a UMI using conditional independence (See Supplementary Methods).

FASTQ files are used as input to the deduping module. The algorithm is written in C and based on a highly efficient and customized data structure. Efficient binary representation of k-mers (up to length 16) enables perfect hashing and rapid computations of hamming distances. The algorithms also make use of BGZF compressed FASTQ files (this is the same compression method used in bam files) which enables indexing and fast random read access within the compressed FASTQ files.

### Trimming Adapters

Fastp (Chen et al., 2018) was implemented with default settings to trim the adapters from reads produced by the QIAseq RNAscan chemistry, with adapter sequences provided in a FASTA file using the--adapter_fasta option.

### Alignment-Based Read Reduction and *De-Novo* Assembly

*De-novo* assembly is a computationally intensive process that has the potential to overwhelm computational resources (Sze et al., 2017). To alleviate this potential bottleneck, a pre-processing step was formulated to reduce the number of identical or high similarity reads entering the *de-novo* assembly stage. A target reference database for read alignment was created using the full nucleotide sequence of the longest coding transcript for all genes targeted by the neuro oncology NGS panel, identified by HGNC (Yates et al., 2017) gene names. BWA-mem (Li and Durbin, 2009) from Sentieon (Sentieon, Mountain View, CA, United States) was used to enable rapid alignment to the gene-based reference database. BWA-mem can be substituted with the open source BWA-mem tool using profile settings for the pipeline. Default parameters for paired-end alignment were used. Best alignments were selected for each read and binned into gene-specific SAM files utilizing the SAMTOOLS (Li et al., 2009) view functionality with the aligned BAM file. A custom python module was developed to identify alignments sharing identical length and chromosomal start and end coordinates and filter the subsequent output to five copies or fewer. Reads passing this stage were output in FASTA format for *de-novo* assembly. Following alignment-based reduction, reads were assembled using the CAP3 (Huang and Madan, 1999) *de-novo* assembly tool with default settings (Supplementary Table S1). Assembly was performed individually on each gene-specific SAM file to improve the specificity of the *de-novo* assembly.

### Aligning Contigs to the Reference Genome

*De-novo* assembled contigs were aligned to the human reference genome (hg19) using BLAT (Kent, 2002). Those generating multiple non-contiguous alignments were retained as potential fusion candidates or splice-variants for downstream characterization. The parameters for BLAT are specified in Supplementary Table S1. Custom modules were developed to remove multiple contigs mapping to identical genomic regions from BLAT results, reducing redundancy in the putative gene fusion breakpoints.

### Filtering Modules

Filtering modules were developed to reduce false positive calls occurring due to mononucleotide repeats, homology, and other recurrent artifacts. To filter the false positives due to mononucleotide repeats, the filtering script looks for repetitive regions using a Mononucleotide Repeat Ratio (MRR). The mononucleotide repeat ratio calculation is described in the Supplementary Methods.

For filtering highly homologous regions, SeekFusion flags calls that are recurrent in every sample due to homology/low complexity using a blocklist. The blocklist calls are presented in the Supplementary Data. Events involving a single transcript are filtered from the output by default. An inclusion feature is provided to enable detection of clinically relevant single transcript events, such as epidermal growth factor receptor variant III (EGFRvIII) in glioblastoma (An et al., 2018).

### Annotation of Contigs Representing Potential Fusions

A custom script was developed to annotate identified contig breakpoints and provide genomic context. Annotations generated include the gene, exon and coding frame status of the fusion, i.e., if the fusion is likely protein-coding. One of the key annotations provided by the annotation module is if a fusion is In-frame, which implies that there was no frame shift in the 3′-gene, regardless of single amino acid mutation or insertion events at the fusion junction. Table 2 contains examples of fusion annotations generated by the pipeline.

**TABLE 2:** Fusion annotation generated by the pipeline for documented fusions.

Fusion	Fusion location	Frame status	5′ exon annotation	3′_exon_annotation
FGFR3-TACC3	Exon-exon_boundary	In-frame	+|End_E17|FGFR3|NM_001163213	+|Start_E11|TACC3|NM_006342
FN1-FGFR1	Exon-exon_boundary	In-frame	-|End_E27|FN1|NM_212482	-|Start_E5|FGFR1|NM_023110
ETV6-NTRK3	Exon-exon_boundary	In-frame	+|End_E5|ETV6|NM_001987	-|Start_E15|NTRK3|NM_001012338
SSX1-SSX8	Exon-exon_boundary	In-frame	+|End_E5|SSX1|NM_005635	+|Start_E6|SSX8|NR_027250

### Putative Fusion Reference Generation and Alignment-Based Event Confirmation

**FIGURE 3:**
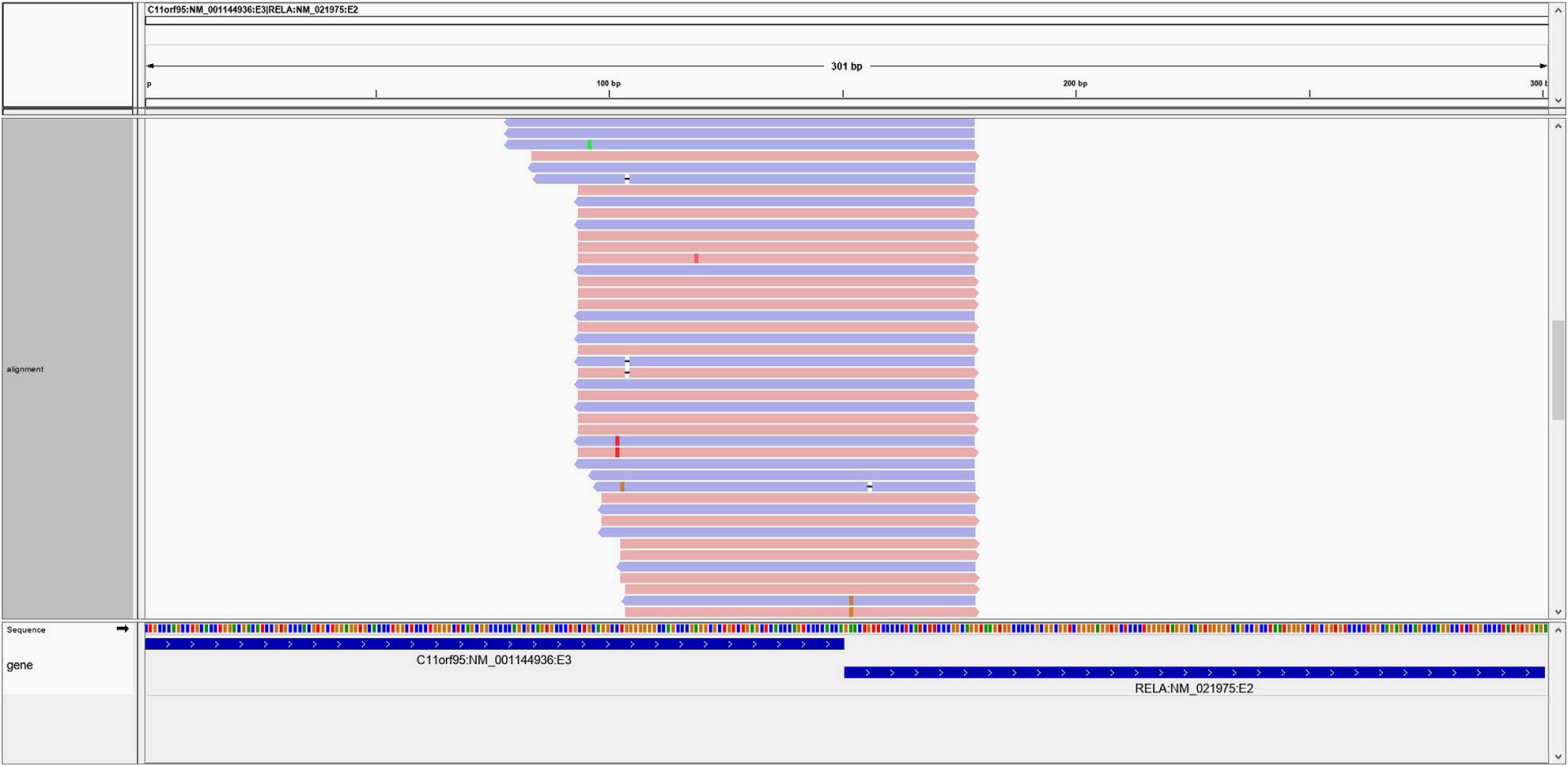
An example of the C11ORF95-RELA positive fusion on IGV. Multiple reads generate high-confidence alignments and span the breakpoint in support of the putative fusion transcript.

All reads originally passing the UMI-based deduping and adapter trimming steps were retrieved and aligned to the hg19 human reference transcriptome from Ensembl and Refseq using BWA-mem. Reads that produced perfect alignments to known gene transcripts were removed from further consideration as they likely represent normal transcriptional events. For each gene fusion candidate identified by the alignment of assembled contigs to the human genome, a chimeric construct of the purported fusion sequence was created (150 base pairs bidirectional beyond the putative breakpoint). Constructs annotated as occurring within a single known transcript were removed from further consideration, with the exception of select known aberrant single-gene events. The retained reads were aligned against the potentially chimeric gene fusion reference sequences using BWA-mem. Only reads spanning at least 10 bases beyond a breakpoint were considered as evidence of a potential fusion. A single supporting read was required as evidence of a fusion candidate and all candidates were output in a standardized variant call format (VCF) file. While minimal read support was required to facilitate downstream benchmarking, the supporting read threshold is user configurable. Finally, the fusion-construct aligned BAM file was used to create an Integrative Genomics Viewer (IGV) (Robinson et al., 2011) session to enable visual inspection of the evidence supporting each fusion candidate (Figure 3).

### Packaging of Pipeline for General Use

The pipeline is available in a Docker container (Docker, 2014) image and is available to download from https://hub.docker.com/repository/docker/jagadhesh89/seekfusion. The usage details are explained in the README file. In addition to the easy to use docker image, the source code is also made available via github (https://github.com/jagadhesh89/seekfusion).

### Benchmarking

Benchmarking was performed for SeekFusion, JAFFA-hybrid (Davidson et al., 2015), STAR-Fusion (Haas et al., 2019) and TOPHAT-Fusion (Kim and Salzberg, 2011), mirroring benchmarking analyses performed by the STAR-Fusion study (Haas et al., 2019). Selected samples were processed through the four pipelines for benchmarking in two separate analyses. In Analysis 1, FASTQs were trimmed with FASTP (Chen et al., 2018), subsequently deduped using UMIs, and supplied as inputs to the pipelines. In Analysis 2, the raw FASTQs were provided as pipeline inputs for the 12 gene fusion positive cases, the 9 gene fusion negative cases, and the positive controls. Analysis 2 was used to assess tool performance with raw sample FASTQs, without deduping, however the adapters and UMIs were trimmed to prune the reads for specific tools (see Supplementary Section S2.5). In Analysis 2, JAFFA-Hybrid posed a scalability challenge; the pipeline ran for over 24 h per sample before failure due to memory requirements. Due to the scalability issue, JAFFA-Hybrid was excluded from Analysis 2. However for the analysis 2, we benchmarked JAFFA-Direct to circumvent the scalability issues of JAFFA-hybrid.

Accuracy was measured based on putative gene fusion calls using the following equation at varying levels of fusion read support thresholds.

$$Accuracy=\frac{True\;Positive+True\;Negative}{True\;Positive+True\;Negative+False\;Positive+False\;Negative}$$

$$\mathrm{Algorithm sensitivity was also measured using}\;True\;Positive\;rate=\frac{True\;Positive}{True\;Positive+False\;negative}$$

For in-silico generated fusions, supporting reads are a known quantity. Mean Absolute Percentage Error (de Myttenaere et al., 2016) (MAPE) and Symmetric Mean Absolute Percentage Error (Tofallis, 2015) (SMAPE) values were calculated as a measure of known supporting reads detected by the four algorithms. A Wilcoxon test was performed on the SMAPE values between SeekFusion and other tools. Key considerations on determining the winning tool from benchmarking involved high accuracy, high true positive rate, ease of installation, ease of usage in a clinical setting and turnaround time for the pipeline.

### Clinical Testing on Neurological Oncology and Sarcoma Samples

The neurological oncology assay was developed, verified, validated, New York (NY) State approved and implemented clinically using the SeekFusion algorithm. In the verification study, 63 total unique samples were processed (59 CNS tumor samples, 1 CNS tumor sample spiked with 13 known synthetic oligonucleotides and 3 brain gliosis samples). The NGS assay results were confirmed by RT-PCR and CMA tests. The overall accuracy of the NGS assay was 96.1% and no fusion transcripts were detected in negative control samples indicating an overall specificity of 100%. Following the clinical go-live of the assay, 2,979 clinical patient samples ordered for neurological oncology testing and were processed using the SeekFusion algorithm. A sarcoma assay was developed, verified, validated and approved for clinical testing by NY state. The assay uses the SeekFusion algorithm and evaluates 138 gene targets for presence of somatic gene fusions and common BCOR internal tandem duplications (ITDs) using the same QIAseq RNAscan chemistry. In the verification study, this next-generation sequencing (NGS) assay was performed in 111 sarcoma formalin-fixed, paraffin-embedded (FFPE) and cytology samples (86 fusion positive and 25 fusion negative). The NGS assay results were confirmed by RT-PCR and FISH tests. The overall accuracy of the NGS assay was 95.5%. No targeted gene fusions were detected in 20 negative control samples (100% specificity), A total of 1,505 clinical cases were processed using the SeekFusion algorithm. An IRB request was submitted and approved to share the clinically reported gene fusions.

## Results

### Accuracy and Sensitivity-Based Performance

**FIGURE 4:**
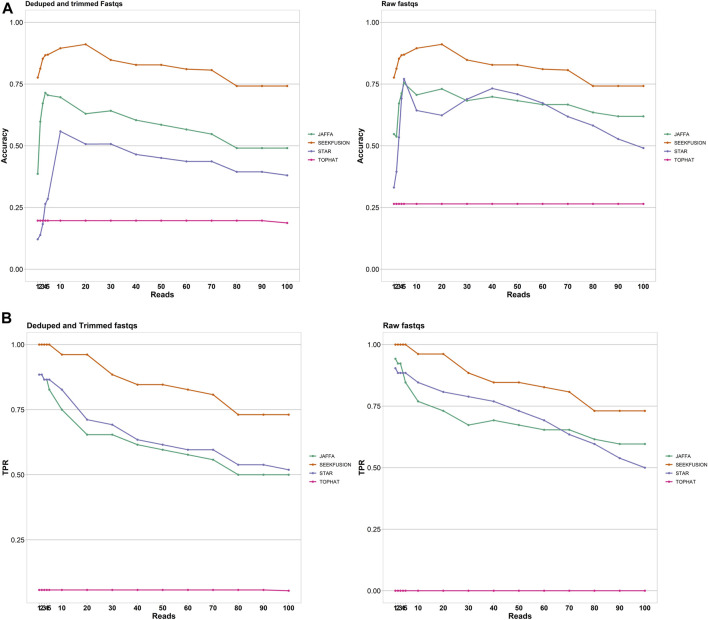
**(A)** Accuracy of the tools was assessed for the tools JAFFA, STAR-Fusion, Tophat Fusion and SeekFusion at varying read support thresholds. SeekFusion demonstrated highest accuracy in detecting the gene fusions at all levels of read support. The median accuracy of SeekFusion was 82% followed by JAFFA-Hybrid at 60%, STAR-Fusion at 39% and Tophat-Fusion at 20% for Mode1. SeekFusion was the only pipeline that demonstrated high accuracy in detecting fusions from raw FASTQs. **(B)** True Positive Rate of the tools was assessed for the tools JAFFA, STAR-Fusion, Tophat Fusion and SeekFusion at varying read support thresholds. SeekFusion demonstrated the highest sensitivity in detecting the gene fusions at all levels of read support. For the deduped and trimmed FASTQs, we observed that the median true positive rate of SeekFusion was 89% followed by STAR-Fusion at 69% and JAFFA-Hybrid at 65%. Tophat Fusion performed least favorably among the tools assessed with a median true positive rate of 1%. SeekFusion was the only tool that successfully detected true positives in the raw FASTQs.

The accuracy of SeekFusion was highest among the benchmarked tools, followed by JAFFA-hybrid, STAR-Fusion and TOPHAT-Fusion (Figure 4A). True positive rate was measured as a surrogate of clinical utility and SeekFusion consistently performed best among the tools at all levels of read support cut-off (Figure 4B). Read support cut-off is commonly applied to fusion detection algorithms for reducing false positive rate and improving accuracy (Liu et al., 2016). Read cut-offs for calling a fusion were assessed for all tools at levels of 1, 2, 3, 4, 5, 10, 20, 30, 40, 50, 60, 70, 80, 90 and 100 supporting reads, and accuracy and true-positive rates were calculated. Intuitively if an algorithm consistently reports gene fusions with fewer reads supporting the event, a higher read cut-off will result in a lower accuracy and true positive rate. JAFFA hybrid was ranked second, followed by STAR-Fusion; however, both JAFFA and STAR-Fusion produced higher numbers of calls beyond the known fusion events. These calls were closely examined and are documented in Supplementary File S1. Manual curation was performed, and the extra calls were classified as false positives; all these calls were due to homology, low complexity, or identified with very low frequency (Supplementary Table S5). Tophat Fusion performed the worst, with a high rate of false negatives. SeekFusion was the only tool observed to be 100% concordant with the expected positive gene fusions whereas STAR-Fusion, JAFFA-Hybrid and TOPHAT-Fusion detected 83, 77 and 1% respectively.

### Levels of Read Support for Detected Fusions

**FIGURE 5:**
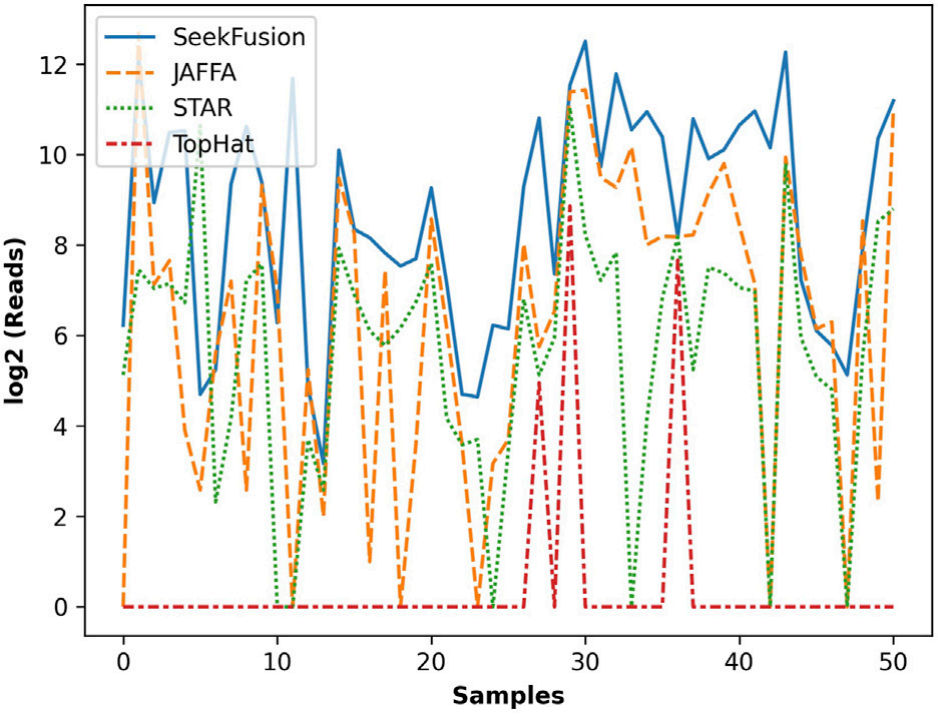
The gene fusion read support in log scale for each fusion detected by SeekFusion, STAR-Fusion, Tophat Fusion and JAFFA. In 83% of cases, SeekFusion demonstrated the highest read support amongst the algorithms tested.

The number of gene fusion-supporting reads detected by the four tools was assessed for the true positive gene fusions. Deduped and trimmed FASTQs were utilized in the analysis since only SeekFusion demonstrated the ability to successfully detect the true positive fusions from raw FASTQs. SeekFusion reported more gene fusion supporting reads than the other tools in 83% of cases (Figure 5). SeekFusion was followed by JAFFA, STAR-Fusion and Tophat Fusion for the number of fusion supporting reads.

MAPE and SMAPE are widely used measures of forecast accuracy; they aid in assessing accuracy based on percentage errors from the actual. MAPE (de Myttenaere et al., 2016) and SMAPE (Tofallis, 2015) values indicate that SeekFusion performed the best in terms of observed to expected gene fusion-supporting reads amongst the four tools (Table 3). A Wilcoxon test (Wilcoxon, 1945) on the SMAPE values between SeekFusion and other tools supported the hypothesis that SeekFusion retrieved the highest proportion of known fusion-supporting reads in the in-silico samples (Table 3). Each tool considered has a different method of fusion transcript identification and these account for differences in levels of read support.

**TABLE 3:** MAPE and SMAPE values for the 4 tools and the *p*-values based on the Wilcoxon test on SMAPE values between SeekFusion and other tools.

Tool	MAPE	SMAPE	*p*-value from Wilcoxon test on SMAPE values between SeekFusion and other tools
SeekFusion	0.21077	0.12887	Not applicable
STAR-fusion	0.78855	0.67441	1.490116e-08
TOPHAT-fusion	1	1	1.490116e-08
JAFFA hybrid	0.54454	0.47727	0.0009347796

### Computational Time Requirements and Peak Memory Utilization

**FIGURE 6:**
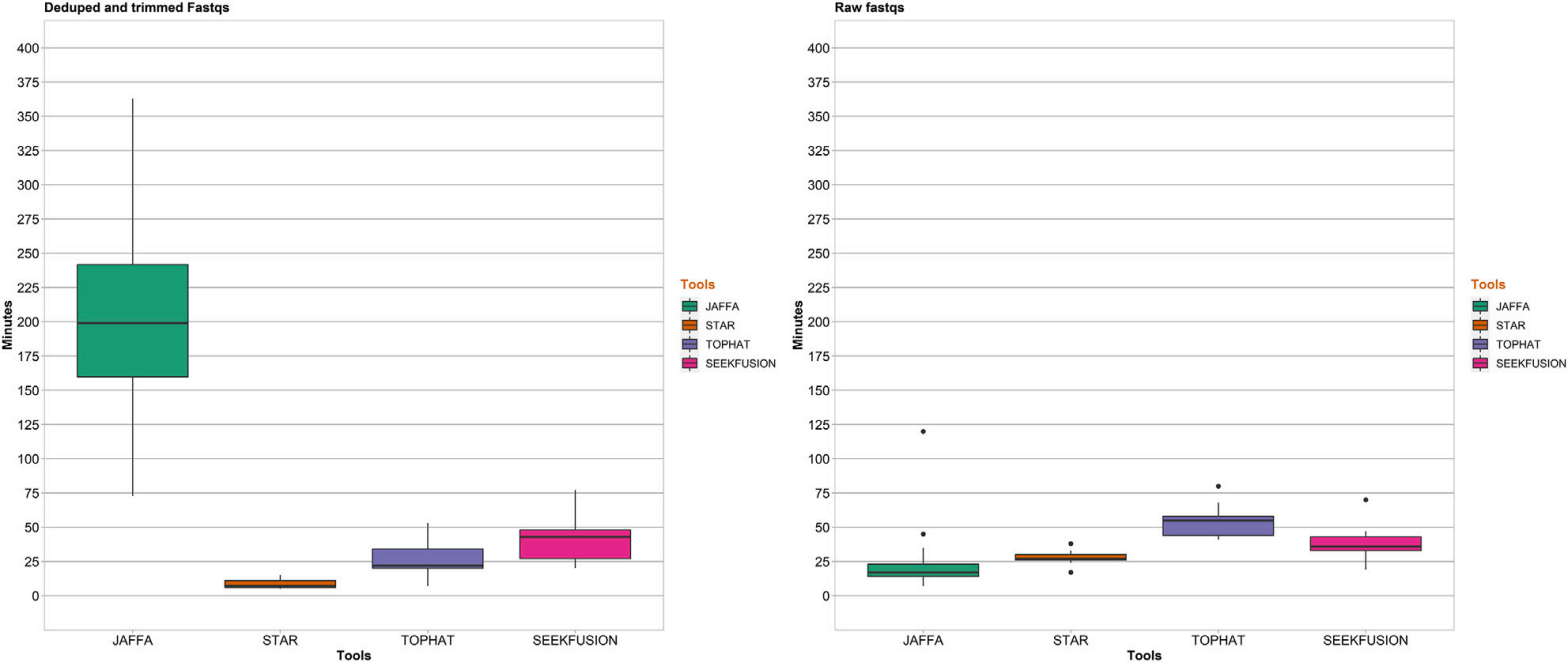
Pipeline completion time in minutes for the 4 tools. For Analysis 1, STAR-Fusion was the fastest among the 4 tools with median completion time of 6 min followed by Tophat Fusion at 20 min, SeekFusion at 45 min and JAFFA-Hybrid at 196 min. For Analysis 2, STAR-Fusion had a median completion time of 29 min, SeekFusion had a median completion time of 36 min, Tophat-Fusion had a median completion time of 53 min and JAFFA-Hybrid exhibited scalability issues withe pipeline failing due to memory issues after running for over 24 h (so the data was capped at 400 min for JAFFA Hybrid in the raw FASTQ mode). To circumvent the scalability issues, analysis in direct mode of JAFFA was performed and this mode demonstrated the fastest median completion time of 20 min.

In addition to analytical performance, clinical bioinformatics pipelines are required to perform in a time-efficient manner to maintain necessary assay turnaround time. Benchmarking of runtimes was performed to assess the suitability of the algorithms for real-world clinical use. The single in-silico sample was excluded from the benchmarking since it does not represent a true patient sample. For both Analysis 1, JAFFA exhibited time-requirements that exceeded all other tools (Figure 6). The remaining three tools were comparable in their runtimes and were all considered amenable to routine clinical use, with STAR-Fusion performing most favorably in terms of time-requirements alone. In Analysis 2 due to scalability challenges posed by JAFFA-hybrid mode, analysis was performed in JAFFA-Direct mode and it was found that JAFFA-Direct mode was most favorable in terms of time-requirements alone.

Peak memory utilization of the individual tools benchmarked were assessed. The maximum input memory for each of the pipelines were capped at 180 GB. For each sample, the maximum memory used by the tool is used for calculating a per tool median memory utilization for analysis 1 and analysis 2. The median is rounded to the nearest GB. JAFFA utilized the most memory with a median memory utilization of 171 GB in analysis 1 and 172 GB in analysis 2. SeekFusion peaked at a median memory of 58 GB in analysis 1 and 64 GB in analysis 2, with CAP3 assembly being the most memory consuming module in the SeekFusion pipeline. STAR-Fusion peaked at a median memory of 30 GB in analysis 1 and 34 GB in analysis 2. Tophat-Fusion used the least memory; with peak of median memory in analysis 1 and analysis 2 of 2 GB.

Taken together, while considering high accuracy, high true positive rate, ease of installation, ease of usage in a clinical setting and turnaround time for the pipeline, SeekFusion was identified as the method for use of clinical assay development. Neurological oncology assay and sarcoma assay were developed, verified and approved as detailed in the *Methods*, *Clinical Testing On Neurological Oncology and Sarcoma Samples* section.

### Clinical Testing Using Neurological and Oncology Assays

**FIGURE 7:**
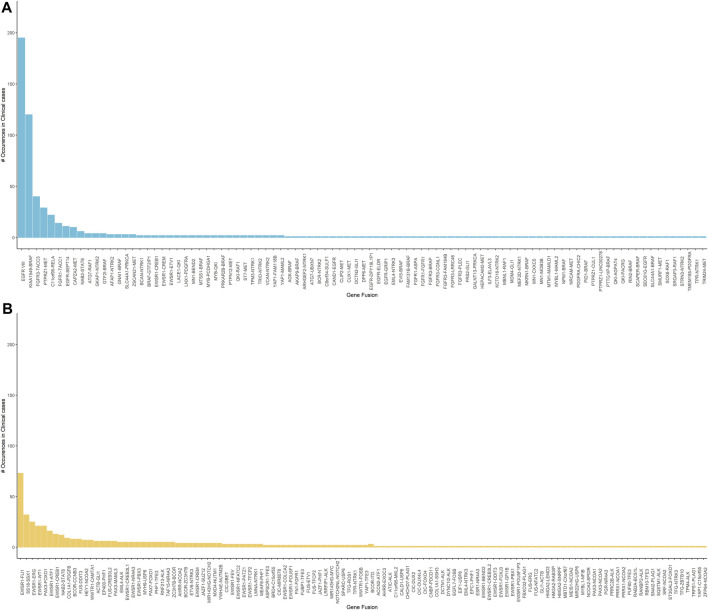
**(A)** Clinically reported gene fusions in neurological oncology cases indicating frequency of common occurring events in the tested cohort. It is observed that the EGFRvIII is the most observed event followed by the KIAA1549-BRAF and the FGFR3-TACC3 fusion. **(B)** Clinically reported gene fusions in sarcoma assay indicating frequency of common occurring events in the tested cohort. EWSR1 related fusions are the most commonly observed in our tested cohort.

Of the 2,979 cases tested clinically using the neurological oncology assay, 569 cases were reported with clinically relevant gene fusions after careful variant review and orthogonal confirmation using RT-PCR (Figure 7A). Out of the 1,505 cases tested clinically using the sarcoma assay, 476 cases were reported (Figure 7B).

In the neurological oncology assay, EGFRvIII was the most commonly found rearrangement, 195 out of 2,979 patients tested were identified with this rearrangement. EGFRvIII has been established to be present in up to 28–30% of the glioblastoma cells and constitutes to be a therapeutic target (Rutkowska et al., 2019). KIAA1549-BRAF was identified in 120 patients and this rearrangement has been established to be frequently found in pilocytic astrocytoma (Chen et al., 2019). In addition, our pipeline picked up several established fusions related to glioblastoma such as FGFR3-TACC3 and PTPRZ1-MET. Confirming the presence of NTRK gene fusions can guide treatment of the solid tumors (Filippi et al., 2021). Using our SeekFusion method on this assay, we reported NTRK gene fusions for 46 cases.

In the sarcoma assay, EWSR1 was the most commonly rearranged gene in our case series, with 3′ partners including FLI1, ERG, WT1, CREB1, ATF, PBX3, CREB3L1, TFCP, NR4A3, NFATC2, POU5F1, DDIT3, CREM, PATZ1, and COLCA2 in a wide variety of tumor types. SS18-SSX1 was reported in 32 synovial sarcoma cases. PAX3 was the 5′ fusion partner in 29 cases with 3′ partners being FOXO1 fusions identified in alveolar rhabdomyosarcoma cases, MAML3 and NCOA1 fusions in two different biphenotypic sinonasal sarcoma cases.

### Novel Fusion Discovery

As described previously the assay chemistry is gene-partner agnostic, enabling identification of novel gene fusions, and many novel fusions were detected in the clinical patient cohort. We have summarized all the neurological cancer and sarcoma gene fusions identified by our method in the Supplementary Table S7. We present two cases here in detail.

**FIGURE 8:**
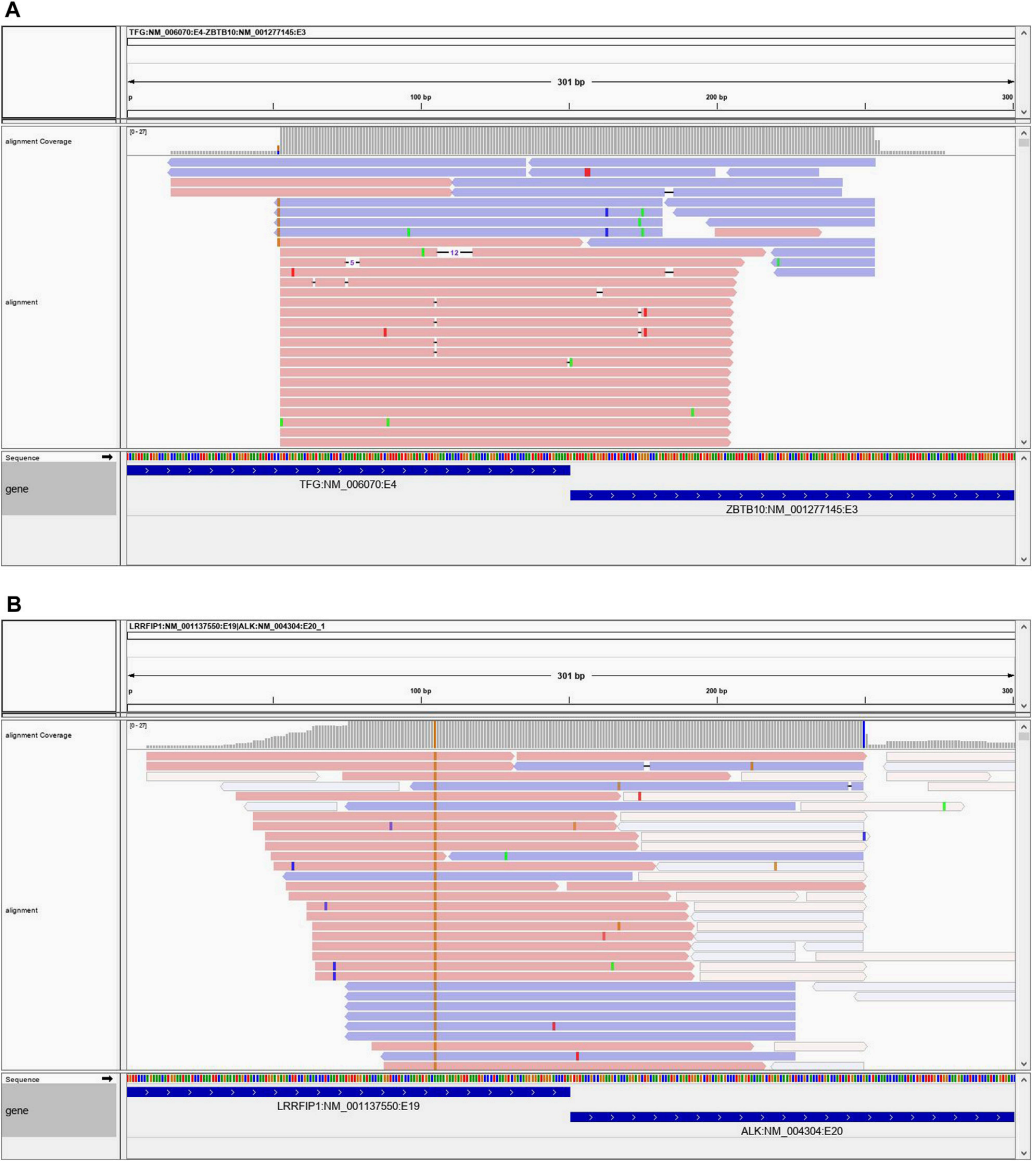
**(A)** Case 1 showing the reads spanning the TFG-ZBTB10 fusion. **(B)** Case 2 showing spanning reads for the LRRFIP1-ALK fusion.

Case 1 is a 45-year-old male who presented with a lower left extremity mass suggestive of a low-grade mesenchymal neoplasm. By immunohistochemistry (IHC) the neoplastic cells were focally positive for desmin, epithelial membrane antigen and STAT6 and showed limited expression of TRK. ALK, OSCAR keratin and TLE1 were negative. Histologically the neoplasm was a very unusual appearing, multicystic proliferation of rather bland small round epithelioid cells, in areas showing the presence of an apparent second cell population, consisting of flattened cells with more abundant eosinophilic cytoplasm. This morphologic appearance was suggestive of at some level an adnexal lesion however a variety of epithelial markers are essentially negative. It also appeared to be related to angiomatoid fibrous histiocytoma although the morphologic features of this lesion are atypical and although it showed very limited coexpression of desmin and EMA it was negative for the EWSR1 and FUS gene fusions that characterize this tumor. Based on the very bland morphology of this lesion and the extremely low mitotic rate it is believed that the lesion is either entirely benign or at most has some limited capacity for local recurrence. NGS testing identified a novel TFG-ZBTB10 fusion (Figure 8A). The specific fusion TFG-ZBTB10 has not been described as a recurrent oncogenic event. However, both genes have been involved in fusion events with several other partners. TFG (Trafficking for Endoplasmic Reticulum to Golgi Regulator) has been reported as a 5′ partner in several fusions genes in acute leukemias, sarcomas and carcinomas. In contrast, ZBTB10 (Zinc Finger and BTB Domain-Containing Protein 10), which encodes for a zinc finger protein, has been found in isolated fusion events with other partner genes in breast and colonic adenocarcinomas.

Case 2 is a 23-year-old male who presented with a 14 × 4.8 × 7.8 cm right chest mass which was eroding the underlying clavicle. Microscopically, this is a spindle cell neoplasm showing a whorling, fascicular growth pattern with scattered chronic inflammatory cells and foci of hyalinized fibrosis. The somewhat enlarged nuclei raise concern for the possibility of a low-grade sarcoma, but they maintain a rather uniform appearance and lack significant atypia. IHC studies show the tumor cells to be positive for FLI-1 and negative for S100 protein, SOX10, desmin, myogenin, WT1, SMA, CD34 and pan-cytokeratin. Given these findings, the differential diagnosis included follicular dendritic sarcoma and angiomatoid fibrous histiocytoma. Additional stains performed locally showed the cells to be negative for CD31, CD35 and CD21. NGS testing revealed a novel RRFIP1-ALK gene fusion (Figure 8B). ALK fusions have been identified in a variety of neoplasms, including inflammatory myofibroblastic tumor, many subtypes of lymphomas and leukemias, adenocarcinomas, benign fibrous histiocytoma, and several other tumors. LRRFIP1 encodes the leucine-rich repeat flightless-interacting protein 1 (FLI-1 interacting protein), a DNA binding transcriptional repressor and may regulate expression of TNF, EGFR and PDGFA. Additionally, it may regulate smooth muscle cell proliferation following arterial damage through PDGFA repression. Previously, an LRRFIP1-MET fusion was identified in an atypical Spitz tumor (PMID: 26013381). There is also another report of an LRRFIP1-FGFR1 fusion in a myeloid neoplasm (PMID: 19369959). While the LRRFIP1-ALK gene fusion observed in this case has not been previously reported in the literature, tumors with ALK fusions have been shown to respond to ALK-inhibitor therapies. Given the histologic findings, it was felt that the tumor was within the spectrum of an inflammatory myofibroblastic tumor.

## Discussion

We have developed a novel bioinformatics pipeline to enable accurate and precise gene fusion detection for commonly used RNA UMI-based amplicon NGS assays. A combination of traditional alignment and *de-novo* assembly-based approaches enables SeekFusion to increase accuracy of fusion calling over other published and widely used gene fusion-calling algorithms, while maintaining reasonable computational expense. We have demonstrated that SeekFusion exhibits analytical sensitivity with minimal false positive calls and produces higher read support per gene fusion call when compared to other benchmarked bioinformatics tools. Furthermore, SeekFusion is computationally efficient and capable of facilitating realistic turnaround times in a clinical setting. The bioinformatics pipeline and quality check turnaround times are less than a day for the neurological oncology assay and the sarcoma assays mentioned in our study, which enables an analytic time of 14 days for the assay, from sample collection, until clinical report.

Although STAR-Fusion, JAFFA-Hybrid and TOPHAT-Fusion have previously been benchmarked to perform adequately for gene fusion detection using standard RNA-Seq assays (Haas et al., 2019), our study demonstrates these tools lack in performance for RNA UMI-based amplicon NGS assays. SeekFusion provides a tailored solution capable of sensitive and specific fusion gene detection from commonly used assays such as QIAseq RNAscan or Archer FusionPlex, thus making it widely applicable for clinical use. SeekFusion pipeline’s applicability to routine clinical use is further aided by its DOCKER-based deployment. Despite the pipeline’s complex multi-step operation, this packaging is compatible with a wide range of deployment systems including high performance clusters and cloud computing platforms. The implementation conceals the minutiae of the pipeline architecture from end-users and ensures ease of configuration with limited requirements for computational expertise.

In terms of computational run-time, all tested algorithms performed at a level that is amenable to clinical turnaround times, with the possible exception of JAFFA, which demonstrated obvious scalability issues and required as much as 6 hours per sample to complete a single analysis. STAR-Fusion, Tophat-Fusion and SeekFusion performed similarly, although STAR-Fusion showed overall lower run-times than the other tools. Considered in totality, however, the overall performance characteristics of SeekFusion combined with clinically favorable run-times demonstrate it to be the optimal solution for fusion transcript identification.

SeekFusion pipeline’s use of common output file formats represent another advantage for its implementation. Most gene fusion calling algorithms utilize arbitrary formats which present compatibility issues with downstream tools. The use of a standard VCF output for candidate events enables more seamless integration with existing tools and workflows. The generation of an IGV session file further facilitates the utilization of SeekFusion’s outputs with common downstream components. The ability to readily visualize the level of support and alignment quality for fusion-supporting reads is a key functionality. The visualization functionality is currently unavailable in the other tested gene fusion calling software and allows end-users to visually judge the quality of the supporting reads and infer confidence of a gene fusion call.

A limitation of the previously published tools profiled in this study, beyond core performance metrics, is the lack of ability to detect clinically relevant single gene events (Supplementary Table S4), such as the EGFR vIII transcript variant that is a relatively frequent event in glioblastomas (An et al., 2018). The ability of SeekFusion to detect aberrant single-gene events expands its clinical utility and offers additional advantages over pre-existing algorithms. Customization of filter settings in the SeekFusion pipeline enables the inclusion of specific single gene events as required by clinical end-users, while maintaining a low rate of false positives by excluding normal transcript variants. Furthermore, we have demonstrated SeekFusion’s ability to detect novel fusions leveraging the gene-partner agnostic Qiaseq RNA, or similar chemistry, further evidencing its clinical utility. It should be noted that SeekFusion has been tailored to utilize the outputs from common PCR and UMI-based RNA assays such as the Qiaseq assay. It is therefore not likely to natively generalize to alternative, clinically utilized, capture-based methods. While these alternative approaches may be fundamentally similar, further customization and benchmarking will be required to assess the potential applicability of SeekFusion to other chemistries and represents a foundation for future development.

A potential limitation of our study was the relatively small number of clinical samples that were available for initial benchmarking. This reflected the difficulties in obtaining broader research consent, which we overcame by supplementing real clinical samples with oligonucleotide spike-ins and in-silico simulated data. Despite the diversity of the test data utilized, SeekFusion demonstrated high analytical sensitivity and specificity across all datatypes when compared to other available tools, further supporting SeekFusion pipeline’s accuracy and versatility. SeekFusion pipeline’s packaging allows it to be installed and deployed in a clinical setting with minimal efforts. The pipeline enables customization of thresholds, tools, and settings through a configuration file. The setup of SeekFusion for another clinical assay is easily enabled through simple user configuration, as mentioned in the readme instructions in https://hub.docker.com/repository/docker/jagadhesh89/seekfusion. To validate SeekFusion and establish analytical sensitivity and specificity for a similar clinical assay, it is recommended to run the pipeline on a robust set of known positives and negatives, including positive in-silico controls and a positive control with spiked in oligonucleotides.

In summary, SeekFusion represents an accurate, time-efficient and versatile solution for the clinical detection of fusion transcripts from common RNA UMI-based amplicon NGS assays. It is our hope that the tool’s public availability and cross-platform capabilities will enable ready deployment and facilitate accurate gene fusion identification in a range of clinical laboratory and disease settings.

## Data Availability

Datasets used for benchmarking analyses in this study is available in the NCBI’s SRA website. The docker shared in article has test samples with real fusions and the entire code base. Any fusion sequence or breakpoint information for identified fusion transcripts in clinical cases can be shared on request. Requests to access the datasets should be directed to balan.jagadheshwar@mayo.edu.
