# Supplementary material for: SeekFusion - A Clinically Validated Fusion Transcript Detection Pipeline for PCR-Based Next-Generation Sequencing of RNA
Source: Front Genet. 2021 Oct 22;12:739054. doi: 10.3389/fgene.2021.739054 (PMC8569241; doi:10.3389/fgene.2021.739054)
Supplement: Supplementary file 1 [file DataSheet1.PDF]

Supplementary Material

1 Supplementary Table

1.1 Supplementary Table 1: Tool and commands for tools

Tool	Command
Cap3	cap3 <gene_reads.fa>
BLAT	blat -stepSize=10 \<br>-repMatch=1024 \<br>-minScore=20 \<br>-minIdentity=100 \<br>-out=blast8 \<br><hg19_reference><br><contigs.fa><br><outputname>
Fastp	fastp <R1.fastq.gz> <R2.fastq.gz>
STAR-Fusion	STAR-Fusion --left_fq ${samplename}_R1.fastq.gz --right_fq ${samplename}_R2.fastq.gz --output_dir star_output --genome_lib_dir GRCh37_gencode_v19_CTAT_lib_Mar012021.plug-n-play/ctat_genome_lib_build_dir –CPU 1
JAFFA	JAFFA-version-1.09/tools/bin/bpipe run JAFFA-version-1.09/JAFFA_hybrid.groovy <SAMPLEFOLDER>/*.fastq.gz
TOPHAT-Fusion	tophat-2.1.0.Linux_x86_64/tophat --bowtie1 -o <tophat_OUTPUT_folder> -p 1 --fusion-search --keep-fasta-order --no-coverage-search -r 0 --mate-std-dev 500 --max-intron-length 100000 --fusion-min-dist 100000 --fusion-anchor-length 13 --fusion-ignore-chromosomes chrM <hg19_reference> <SAMPLE>_R1.fastq.gz <SAMPLE>_R2.fastq.gz

1.2 Supplementary Table 2: Genes targeted in the neurological oncology panel

NFASC	*CLCN6*	*BRAF*	*PCDHGA1*	*MET*	*MKRN1*	*CLIP2*
TFG	*ESR1*	*FXR1*	*NAV1*	*YAP1*	*EWSR1*	*GFI1B*
PKD1	*NRF1*	*EGFR*	*RECK*	*MYB*	*VCL*	*JPX*
NTRK3	*AGBL4*	*GLI1*	*BCAN*	*AFAP1*	*SRGAP3*	*ST6GAL1*
FAM131B	*PDGFRA*	*MAMLD1*	*RELA*	*NDRG1*	*DIP2C*	*UBE2J2*
MYBL1	*ELAVL3*	*MMP16*	*DDX31*	*C11orf95*	*CXXC5*	*FOXR2*
GFI1	*ETV6*	*NAB2*	*FYCO1*	*FGFR3*	*RNF130*	*SEPT14*
BIRC5	*FLI1*	*NTRK2*	*FAM118B*	*KIAA1549*	*PVT1*	
FGFR1	*GNAI1*	*TACC1*	*BTBD1*	*PRKCA*	*BEND2*	
STAT6	*MN1*	*TACC3*	*C8orf34*	*PTPRZ1*		
PCSK5	*MST1R*	*ATG7*	*SLC44A1*	*RAF1*		
TPM3	*MYC*	*QKI*	*NSD2*	*NELFE*		
LINC01420	*NTRK1*	*MACF1*	*NACC2*	*SLIT1*		

1.3 Supplementary Table 3: Gene Targets in Sarcoma assay

ACTB	*AHRR*	*ALK*	*ASPSCR1*	*ATF1*	*ATIC*	*SQSTM1*	*SRSF3*
BCOR	*BRD3*	*BRD4*	*CAMTA1*	*CARS*	*CCNB3*	*SS18*	*STAT6*
CDH11	*CNBP*	*COL1A1*	*COL1A2*	*COL3A1*	*COL6A3*	*TCF12*	*TFE3*

CDX1	*CD63*	*CEP128*	*CIC*	*CITED2*	*CLTC*	*TPM4*	*USP6*
CREB1	*CREB3L1*	*CREB3L2*	*CSF1*	*CXorf67*	*C11orf95*	*WWTR1*	*YWHAE*
DDIT3	*DUX4*	*DVL2*	*EML4*	*EPC1*	*EP400*	*SRF*	*SSX1*
ERG	*ETV1*	*ETV4*	*ETV6*	*EWSR1*	*FEV*	*SS18L1*	*SUZ12*
FGFR1	*FLI1*	*FN1*	*FOSB*	*FOXO1*	*FOXO4*	*TEAD1*	*TFG*
FUS	*GLI1*	*HAS2*	*HEY1*	*HMGA2*	*IRF2BP2*	*TPR*	*VCL*
JAZF1	*KIRREL*	*KLF17*	*LAMTOR1*	*LPP*	*MAML3*	*YAP1*	*ZC3H7B*
MBTD	*MEAF6*	*MED12*	*MIR143HG*	*MKL2*	*MYH9*	*ZFP36*	*ZNF444*
NAB2	*NCOA1*	*NCOA2*	*NFATC2*	*NFIB*	*NOTCH1*	*VGLL2*	*WT1*
NOTCH2	*NR4A3*	*NTRK1*	*NTRK3*	*NUMA1*	*NUTM1*	*SSX2*	*SSX4*
NUTM2B	*OMD*	*OPHN1*	*PATZ1*	*PAX3*	*PAX7*	*S100A10*	*TAF15*
PBX1	*PBX3*	*PDGFB*	*PDPN*	*PHF1*	*PLAG1*	*THRAP3*	*TPM3*
PLPP3	*POU5F1*	*PPFIBP1*	*PRDM10*	*PRKCA*	*PRKCB*		
PRKCD	*RAB2A*	*RAD51B*	*RANBP2*	*RNF213*	*RRAGB*		
SEC31A	*SERPINE1*	*SETBP1*	*SFMBT1*	*SMARCA5*	*SP3*		

1.4 Supplementary Table 4: Common transcript events accounted for reporting by the SeekFusion pipeline

GeneA	GeneA	Chromosome	Start	End

GLI1	*GLI1*	chr12	57854337	57858456
GLI1	*GLI1*	chr12	57857574	57858485
BIRC5	*BIRC5*	chr17	76212862	76218908
PDGFRA	*PDGFRA*	chr4	55133908	55139704
EGFR	*EGFR*	chr7	55229324	55238868
EGFR	*EGFR*	chr7	55228031	55240676
EGFR	*EGFR*	chr7	55087058	55223523
EGFR	*EGFR*	chr7	55268106	55272949
EGFR	*EGFR*	chr7	55268106	55270210
EGFR	*EGFR*	chr7	55229249	55233060
EGFR	*EGFR*	chr7	55087058	55266410
EGFR	*EGFR*	chr7	55211181	55218987
EGFR	*EGFR*	chr7	55233130	55237999
EGFR	*EGFR*	chr7	55177651	55209979
EGFR	*EGFR*	chr7	55087058	55209979
EGFR	*EGFR*	chr7	55219055	55220239
EGFR	*EGFR*	chr7	55221845	55223523
EGFR	*EGFR*	chr7	55238868	55233130

EGFR	*EGFR*	chr7	55270210	55269475
EGFR	*EGFR*	chr7	55270318	55272949
MST1R	*MST1R*	chr3	49933628	49933313
FGFR1	*FGFR1*	chr8	38279315	38271155
FGFR1	*FGFR1*	chr8	38277106	38271149
NELFE	*NELFE*	chr6	31922472	31922345
PCSK5	*PCSK5*	chr9	78547399	78682871
RELA	*RELA*	chr11	65430297	65429559
BCOR	*BCOR*	chrX	39911365	39911457
BCOR	*BCOR*	chrX	39911374	39911466
BCOR	*BCOR*	chrX	39911407	39911493
BCOR	*BCOR*	chrX	39911419	39911529
BCOR	*BCOR*	chrX	39911365	39911457
BCOR	*BCOR*	chrX	39911407	39911493
BCOR	*BCOR*	chrX	39911407	39911496
FGFR1	*FGFR1*	chr8	38271436	38275891

1.5 Supplementary Table 5: Common artifacts in tools

Tool/ pipeline	Call	Reason for false positive

**STAR-Fusion**	CTD-2328D6.1--PCDHGA10	Pseudogene (CTD2328D6.1 is classified as a pseudogene)
**STAR-Fusion**	CTD-2328D6.1--PCDHGA11	Pseudogene (CTD2328D6.1 is classified as a pseudogene)
**STAR-Fusion**	CTD-2328D6.1--PCDHGA12	Pseudogene (CTD2328D6.1 is classified as a pseudogene)
**STAR-Fusion**	CTD-2328D6.1--PCDHGA3	Pseudogene (CTD2328D6.1 is classified as a pseudogene)
**STAR-Fusion**	CTD-2328D6.1--PCDHGA4	Pseudogene (CTD2328D6.1 is classified as a pseudogene)
**STAR-Fusion**	CTD-2328D6.1--PCDHGA5	Pseudogene (CTD2328D6.1 is classified as a pseudogene)
**STAR-Fusion**	CTD-2328D6.1--PCDHGA6	Pseudogene (CTD2328D6.1 is classified as a pseudogene)
**STAR-Fusion**	CTD-2328D6.1--PCDHGA7	Pseudogene (CTD2328D6.1 is classified as a pseudogene)
**STAR-Fusion**	CTD-2328D6.1--PCDHGA8	Pseudogene (CTD2328D6.1 is classified as a pseudogene)
**STAR-Fusion**	CTD-2328D6.1--PCDHGA9	Pseudogene (CTD2328D6.1 is classified as a pseudogene)
**STAR-Fusion**	CTD-2328D6.1--PCDHGB1	Pseudogene (CTD2328D6.1 is classified as a pseudogene)
**STAR-Fusion**	CTD-2328D6.1--PCDHGB2	Pseudogene (CTD2328D6.1 is classified as a pseudogene)
**STAR-Fusion**	CTD-2328D6.1--PCDHGB3	Pseudogene (CTD2328D6.1 is classified as a pseudogene)
**STAR-Fusion**	CTD-2328D6.1--PCDHGB4	Pseudogene (CTD2328D6.1 is classified as a pseudogene)
**STAR-Fusion**	CTD-2328D6.1--PCDHGB6	Pseudogene (CTD2328D6.1 is classified as a pseudogene)
**STAR-Fusion**	CTD-2328D6.1--PCDHGB7	Pseudogene (CTD2328D6.1 is classified as a pseudogene)

**STAR-Fusion**	CTD-2328D6.1--PCDHGC3	Pseudogene (CTD2328D6.1 is classified as a pseudogene)
**STAR-Fusion**	CTD-2328D6.1--PCDHGC4	Pseudogene (CTD2328D6.1 is classified as a pseudogene)
**STAR-Fusion**	CTD-2328D6.1--PCDHGC5	Pseudogene (CTD2328D6.1 is classified as a pseudogene)
**STAR-Fusion**	YAP1--HGS	Homology (The HGS end is identical to another region within transcriptome, not a confident call)
**STAR-Fusion**	SRGAP3--TRAF3	Homology (Part of TRAF3 that is mapped is 100% identical to SRGAP3 region)
**STAR-Fusion**	UHRF1BP1L--QKI	Homology (The UHRF1BP1L is classified as a highly homologous gene and part that maps to this gene is homologous to multiple regions in the transcriptome)
**STAR-Fusion**	AC022201.4--QKI	Homology (The AC022201.4 is a classified LncRNA and the part that maps here is homologous to multiple regions in the transcriptome)
**STAR-Fusion**	JUP--PRKCA	Homology (The JUP part that is mapped is highly identical to to other regions in the transcriptome)
**STAR-Fusion**	PTPRZ1--AACS	Low complexity (The low complexity in this region to PolyT repeats causes this call, as reads map here due to low complexity)
**STAR-Fusion**	QKI--MTA2	Low frequency (0.00025 % frequency)
**STAR-Fusion**	hsamir125a--QKI	Homology (the hsamir125a is classified as a homologous micro RNA)
**STAR-Fusion**	PTPRZ1--FAM115A	Low frequency (0.000009% frequency)
**STAR-Fusion**	SPTBN1--NTRK2	Low frequency (0.00005% frequency)
**STAR-Fusion**	PTPRZ1-VAPA	Low frequency (0.000002% frequency)
**STAR-Fusion**	PTPRZ1--RNY4	Homology (The part that aligns to RNY4 is highly homologous to various regions across transcriptome)
**STAR-Fusion**	YAP1--HGS	Homology (The HGS end is identical to another region within transcriptome)
**STAR-Fusion**	PTPRZ1--RNY1	Homology (The part that aligns to RNY1 is highly homologous to various regions across transcriptome)
**STAR-Fusion**	MCMBP--NTRK2	Low frequency (0.0015%)
**STAR-Fusion**	JPX--KTN1	Low complexity in KTN1 region (KTN1 gene has T and A repeats)
**STAR-Fusion**	PACRG--QKI	Low complexity (PACRG part is highly repetitive)
**STAR-Fusion**	SRGAP3--OXTR	Homology (The read from SRGAP3 also maps to OXTR)
**STAR-Fusion**	MYB--PCDHGA10	Homology (PCDHG family homology call)

**STAR-Fusion**	MYB--PCDHGA11	Homology (PCDHG family homology call)
**STAR-Fusion**	MYB--PCDHGA12	Homology (PCDHG family homology call)
**STAR-Fusion**	MYB--PCDHGA3	Homology (PCDHG family homology call)
**STAR-Fusion**	MYB--PCDHGA4	Homology (PCDHG family homology call)
**STAR-Fusion**	MYB--PCDHGA5	Homology (PCDHG family homology call)
**STAR-Fusion**	MYB--PCDHGA6	Homology (PCDHG family homology call)
**STAR-Fusion**	MYB--PCDHGA7	Homology (PCDHG family homology call)
**STAR-Fusion**	MYB--PCDHGA8	Homology (PCDHG family homology call)
**STAR-Fusion**	MYB--PCDHGA9	Homology (PCDHG family homology call)
**STAR-Fusion**	MYB--PCDHGB1	Homology (PCDHG family homology call)
**STAR-Fusion**	MYB--PCDHGB2	Homology (PCDHG family homology call)
**STAR-Fusion**	MYB--PCDHGB3	Homology (PCDHG family homology call)
**STAR-Fusion**	MYB--PCDHGB4	Homology (PCDHG family homology call)
**STAR-Fusion**	MYB--PCDHGB6	Homology (PCDHG family homology call)
**STAR-Fusion**	MYB--PCDHGB7	Homology (PCDHG family homology call)
**STAR-Fusion**	MYB--PCDHGC3	Homology (PCDHG family homology call)
**STAR-Fusion**	MYB--PCDHGC4	Homology (PCDHG family homology call)
**STAR-Fusion**	MYB--PCDHGC5	Homology (PCDHG family homology call)
**STAR-Fusion**	FAM131B--BRAFP1	Pseudogene (BRAFP1 homologous to BRAF)
**STAR-Fusion**	ING5--QKI	Homology (ING5 part maps to multiple regions with high identity)
**STAR-Fusion**	KIAA1549--CLDN14	Homology (the part that maps to CLDN14 is identical to a part in KIAA1549)
**STAR-Fusion**	RNU4ATAC--RAF1	Homology (the part that maps to RNU4ATAC maps multiply to other parts of transcriptome)
**STAR-Fusion**	EGFR--PARD3	Low frequency (0.00005% frequency)

**STAR-Fusion**	CTD-2328D6.1--QKI	Pseudogene (CTD2328D6.1 is classified as a pseudogene)
**STAR-Fusion**	AC079949.1--BRAF	Low complexity (The AC079949.1 region is GC rich)
**STAR-Fusion**	MAN1A2--QKI	Low complexity (MAN1A2 has low complexity with polyA repeats and there is only one C in the reads aligned to MAN1A2)
**STAR-Fusion**	MOV10L1--NTRK2	Low complexity (MOV10L1 has low complexity with poly T and poly C repeats)
**STAR-Fusion**	QKI--SCD5	Low complexity (polyA repeats)
**STAR-Fusion**	RMRP--QKI	Homology (The RMRP part is highly identical to other regions in transcriptome)
**STAR-Fusion**	PTPRZ1--PSMB1	Low frequency (0.00001%)
**STAR-Fusion**	SMIM4--PCDHGA10	Fusion reads do not span SMIM4 gene for more than 5 bases (PCDHG family homology)
**STAR-Fusion**	SMIM4--PCDHGA11	Fusion reads do not span SMIM4 gene for more than 5 bases (PCDHG family homology)
**STAR-Fusion**	SMIM4--PCDHGA12	Fusion reads do not span SMIM4 gene for more than 5 bases (PCDHG family homology)
**STAR-Fusion**	SMIM4--PCDHGA3	Fusion reads do not span SMIM4 gene for more than 5 bases (PCDHG family homology)
**STAR-Fusion**	SMIM4--PCDHGA4	Fusion reads do not span SMIM4 gene for more than 5 bases (PCDHG family homology)
**STAR-Fusion**	SMIM4--PCDHGA5	Fusion reads do not span SMIM4 gene for more than 5 bases (PCDHG family homology)
**STAR-Fusion**	SMIM4--PCDHGA6	Fusion reads do not span SMIM4 gene for more than 5 bases (PCDHG family homology)
**STAR-Fusion**	SMIM4--PCDHGA7	Fusion reads do not span SMIM4 gene for more than 5 bases (PCDHG family homology)
**STAR-Fusion**	SMIM4--PCDHGA8	Fusion reads do not span SMIM4 gene for more than 5 bases (PCDHG family homology)
**STAR-Fusion**	SMIM4--PCDHGA9	Fusion reads do not span SMIM4 gene for more than 5 bases (PCDHG family homology)
**STAR-Fusion**	SMIM4--PCDHGB1	Fusion reads do not span SMIM4 gene for more than 5 bases (PCDHG family homology)
**STAR-Fusion**	SMIM4--PCDHGB2	Fusion reads do not span SMIM4 gene for more than 5 bases (PCDHG family homology)
**STAR-Fusion**	SMIM4--PCDHGB3	Fusion reads do not span SMIM4 gene for more than 5 bases (PCDHG family homology)
**STAR-Fusion**	SMIM4--PCDHGB4	Fusion reads do not span SMIM4 gene for more than 5 bases (PCDHG family homology)
**STAR-Fusion**	SMIM4--PCDHGB6	Fusion reads do not span SMIM4 gene for more than 5 bases (PCDHG family homology)
**STAR-Fusion**	SMIM4--PCDHGB7	Fusion reads do not span SMIM4 gene for more than 5 bases (PCDHG family homology)

**STAR-Fusion**	SMIM4--PCDHGC3	Fusion reads do not span SMIM4 gene for more than 5 bases (PCDHG family homology)
**STAR-Fusion**	SMIM4--PCDHGC4	Fusion reads do not span SMIM4 gene for more than 5 bases (PCDHG family homology)
**STAR-Fusion**	SMIM4--PCDHGC5	Fusion reads do not span SMIM4 gene for more than 5 bases (PCDHG family homology)
**STAR-Fusion**	THBS1--PCDHGA10	Fusion reads do not span THBS1 gene for more than 5 bases (PCDHG family homology)
**STAR-Fusion**	THBS1--PCDHGA11	Fusion reads do not span THBS1 gene for more than 5 bases (PCDHG family homology)
**STAR-Fusion**	THBS1--PCDHGA12	Fusion reads do not span THBS1 gene for more than 5 bases (PCDHG family homology)
**STAR-Fusion**	THBS1--PCDHGA3	Fusion reads do not span THBS1 gene for more than 5 bases (PCDHG family homology)
**STAR-Fusion**	THBS1--PCDHGA4	Fusion reads do not span THBS1 gene for more than 5 bases (PCDHG family homology)
**STAR-Fusion**	THBS1--PCDHGA5	Fusion reads do not span THBS1 gene for more than 5 bases (PCDHG family homology)
**STAR-Fusion**	THBS1--PCDHGA6	Fusion reads do not span THBS1 gene for more than 5 bases (PCDHG family homology)
**STAR-Fusion**	THBS1--PCDHGA7	Fusion reads do not span THBS1 gene for more than 5 bases (PCDHG family homology)
**STAR-Fusion**	THBS1--PCDHGA8	Fusion reads do not span THBS1 gene for more than 5 bases (PCDHG family homology)
**STAR-Fusion**	THBS1--PCDHGA9	Fusion reads do not span THBS1 gene for more than 5 bases (PCDHG family homology)
**STAR-Fusion**	THBS1--PCDHGB1	Fusion reads do not span THBS1 gene for more than 5 bases (PCDHG family homology)
**STAR-Fusion**	THBS1--PCDHGB2	Fusion reads do not span THBS1 gene for more than 5 bases (PCDHG family homology)
**STAR-Fusion**	THBS1--PCDHGB3	Fusion reads do not span THBS1 gene for more than 5 bases (PCDHG family homology)
**STAR-Fusion**	THBS1--PCDHGB4	Fusion reads do not span THBS1 gene for more than 5 bases (PCDHG family homology)
**STAR-Fusion**	THBS1--PCDHGB6	Fusion reads do not span THBS1 gene for more than 5 bases (PCDHG family homology)
**STAR-Fusion**	THBS1--PCDHGB7	Fusion reads do not span THBS1 gene for more than 5 bases (PCDHG family homology)
**STAR-Fusion**	THBS1--PCDHGC3	Fusion reads do not span THBS1 gene for more than 5 bases (PCDHG family homology)
**STAR-Fusion**	THBS1--PCDHGC4	Fusion reads do not span THBS1 gene for more than 5 bases (PCDHG family homology)
**STAR-Fusion**	THBS1--PCDHGC5	Fusion reads do not span THBS1 gene for more than 5 bases (PCDHG family homology)
**STAR-Fusion**	GOLGA3--QKI	Low complexity (GA repeats near breakpoint)

**STAR-Fusion**	RP11-317B7.2--STAT6	Low complexity (Poly A repeats)
**STAR-Fusion**	SRGAP3--AC010642.1	Homology (The AC010642.1 is homologous to a zinc finger motif that has repeats)
**STAR-Fusion**	SRGAP3--ZNF8	Homology (part of read mapped to SRGAP3 also maps to ZNF8 due to homology)
**STAR-Fusion**	BCAN--PGLS	Low complexity (The PGLS part has lot of GC bases)
**STAR-Fusion**	GS1-165B14.2--QKI	Low complexity (The GS1-165B14.2 part has lot pf polyA repeats)
**STAR-Fusion**	KIAA1549--EVX2	Low complexity (EVX2 part has lot of T repeats)
**STAR-Fusion**	QKI--AL078585.1	Homology (The AL078585.1 part is identical to another region in transcriptome)
**STAR-Fusion**	ARSG--PDGFRA	Low complexity (noisy alignments)
**STAR-Fusion**	EGFR--ZFP36L2	Low complexity (The ZFP36L2 gene has lot of GC content)
**STAR-Fusion**	FGFR1--PILRB	Low complexity (repetitive region in PILRB side)
**STAR-Fusion**	PTPRZ1--RN7SL674P	Homology (The RN7SL674P maps to many regions across transcriptome)
**STAR-Fusion**	PTPRZ1--SNHG16	Low complexity (noisy alignments)
**STAR-Fusion**	QKI--LARP4B	Homology (The LARP4B part maps to multiple regions across transcriptome)
**STAR-Fusion**	RP11-168O16.1--QKI	Low complexity (RP11-168O16.1 has many repeats)
**STAR-Fusion**	SNORD119--FGFR1	Low complexity (polyA repeats)
**STAR-Fusion**	SULT1C2--BRAF	Low complexity (The SULT1C2 region has poly A repeats)
**STAR-Fusion**	TPM3--MRPS21	Homology (The regions mapped to TPM3 and MRPS21 map multiple to other regions in transcriptome)
**STAR-Fusion**	ETV6--METTL25	Homology (The ETV6 first few bases are homologous to other regions in transcriptome)
**STAR-Fusion**	HNRNPK--NTRK2	Homology (The HNRNPK part multiply maps to other regions in transcriptome)
**STAR-Fusion**	SEZ6--NTRK2	Homologous regions and low complexity (The SEZ6 part of mapping is homologous to NTRK2 and due to low complexity its mapping)
**STAR-Fusion**	SLC44A1--ACIN1	Low complexity (ACIN1 gene has A and G repeats)
**STAR-Fusion**	SLC6A4--PRKCA	Homology (SLC6A4 part is homologous to PRKCA gene part leading to false call)

**STAR-Fusion**	CTD-2206N4.4--AC016773.1	Pseudogene (CTD-2206N4.4 is a classified as a pseudogene)
**STAR-Fusion**	CTD-2206N4.4--TACC3	Pseudogene (CTD-2206N4.4 is a classified as a pseudogene)
**STAR-Fusion**	AC011525.2--QKI	Homology (AC011525.2 part maps multiply to other regions)
**STAR-Fusion**	RAB2A--QKI	Homology (RAB2A part maps multiple to other regions)
**STAR-Fusion**	SRGAP3--AC010642.1	Homology (Part of AC010642.1 maps identical to SRGAP3 end)
**STAR-Fusion**	QKI--APP	Homology (Part of APP maps to other regions within transcriptome)
**STAR-Fusion**	KIAA1549--EGFR	Low complexity (The KIAA1549 is repetitive)
**STAR-Fusion**	MIR144--QKI	Low complexity (The MIR144 part is repetitive with poly A and poly T repeats)
**STAR-Fusion**	SRGAP3--RECQL4	Low frequency (0.02%)
**STAR-Fusion**	BCAN--SOX9	Low complexity (Lot of C repeats on either ends)
**STAR-Fusion**	CTD-3148I10.1--EGFR	Noisy alignments
**STAR-Fusion**	EGFR--AC008265.2	Pseudogene (AC008265.2 is classified as a pseudogene)
**STAR-Fusion**	EGFR--RTN4	Low complexity (RTN4 has poly A repeats)
**STAR-Fusion**	FGFR1--MALAT1	Low complexity (Highly repetitive MALAT1 region causing multiple mappings)
**STAR-Fusion**	HHIP--EGFR	Low complexity (HHIP part of mapping has lot of GA repeats)
**STAR-Fusion**	QKI--CASK	Low complexity (The CASK part has a lot of poly A and T repeats)
**STAR-Fusion**	CTD-2328D6.1--RAF1	Pseudogene (CTD2328D6.1 is classified as a pseudogene)
**STAR-Fusion**	IGF2--RELA	Noisy alignments
**STAR-Fusion**	AC009133.14--RELA	Low complexity (AC009133.14 part is highly repetitive)
**STAR-Fusion**	BCAN--DMD	Low complexity and Homology (DMD part has low complexity and hence aligns multiply across transcriptome)
**STAR-Fusion**	EGFR--PRMT2	Low complexity (PRMT2 gene has a lot of G repeats)
**STAR-Fusion**	IGF2--FGFR1	Homology (IGF2 maps multiply to many regions in transcriptome)

**STAR-Fusion**	RP11-586K2.1--PRKAR1A	Noisy alignments
**STAR-Fusion**	NF1--PRKCA	Low frequency (0.01%)
**STAR-Fusion**	CTD-2328D6.1--NTRK2	Pseudogene (CTD2328D6.1 is classified as a pseudogene)
**STAR-Fusion**	RP11-586K2.1--APOD	Homology (The RP11-586K2.1 maps identical to other parts of transcriptome)
**JAFFA-Hybrid**	NAV1:MT-RNR2	Noisy alignments
**JAFFA-Hybrid**	OLFM2:PCDHGA1	Low complexity (OLFM2 part has multiple mappings to other parts of transcriptome due to homology)
**JAFFA-Hybrid**	SPTBN1:NTRK2	Low frequency (0.0008%)
**JAFFA-Hybrid**	RNF144A:PCDHGA1	Homology (The RNF144A part maps multiple to other regions of transcriptome)
**JAFFA-Hybrid**	DHFR:NTRK2	Noisy alignments with insertion and also homology of DHFR aligned part to other regions
**JAFFA-Hybrid**	QKI:MT-RNR2	Noisy spanning reads with insertion and reads do not span a lot of bases
**JAFFA-Hybrid**	QKI:CREBZF	Low frequency (0.00001%)
**JAFFA-Hybrid**	PTPRZ1:VAPA	No spanning reads in alignment to fusion construct
**JAFFA-Hybrid**	PTPRZ1:MT-RNR2	No spanning reads in alignment to fusion construct
**JAFFA-**	NTRK2:MT-RNR2	Noisy spanning reads and reads do not span a lot of bases

**Hybrid**		
**JAFFA-Hybrid**	MYO7A-PCDHGA1	Homology (Part of read in PCDGH maps multiply to other regions in transcriptome)
**JAFFA-Hybrid**	XPNPEP3-PCDHGA1	Homology (Part of read in PCDGH maps multiply to other regions in transcriptome)
**JAFFA-Hybrid**	RTN4-NTRK3	Low frequency (0.002%)
**JAFFA-Hybrid**	ATG7-VGLL4	Low frequency (0.02%)
**JAFFA-Hybrid**	ATG7-TSEN2	Homology (the TSEN2 part aligns multiply to other regions of transcriptome)
**JAFFA-Hybrid**	ATG7-CLASP2	Homology (the CLASP2 part aligns multiply to other regions of transcriptome)
**JAFFA-Hybrid**	LINC00886-PCDHGA1	Low frequency and LINC00886 is non protein coding RNA (0.00003%)
**JAFFA-Hybrid**	SRGAP3-OXTR	Homology (The read from SRGAP3 also maps to OXTR)
**JAFFA-Hybrid**	PDGFRA-MT-RNR2	No spanning reads in alignment to fusion construct
**JAFFA-Hybrid**	PCDHA3-PCDHGA1	Homology (PCDH family)
**JAFFA-Hybrid**	RNF130-DDX46	Homology (RNF part is homologous to other regions in transcriptome)

**JAFFA - Hybrid**	CTD-2007H13.3-PCDHGA1	Pseudogene (CTD-2007H13.3 is a processed pseudogene)
**JAFFA - Hybrid**	PACRG-QKI	Low frequency (0.00003%)
**JAFFA - Hybrid**	QKI-MT-RNR1	Noisy spanning reads with insertion and reads do not span a lot of bases
**JAFFA - Hybrid**	PTPRZ1-RNY4	Homology (The part that aligns to RNY1 is highly homologous to various regions across transcriptome)
**JAFFA - Hybrid**	PTPRZ1-RNY3	Homology (The part that aligns to RNY3 is highly homologous to various regions across transcriptome)
**JAFFA - Hybrid**	PTPRZ1-RNY1	Homology (The part that aligns to RNY4 is highly homologous to various regions across transcriptome)
**JAFFA - Hybrid**	PTPRZ1-KIAA2026	Low complexity (KIAA2026 is highly repetitive)
**JAFFA - Hybrid**	VOPP1-EGFR	Homology (VOPP1 part is 100% identical to other regions of transcriptome)
**JAFFA - Hybrid**	FGFR1-AHSA2	Low complexity (The part of AHSA2 is composed of repeats)
**JAFFA - Hybrid**	LINC00476-PCDHGA1	Low freqeuncy (0.002%) and LINC00476 is non protein coding RNA
**JAFFA - Hybrid**	MT-RNR2-NTRK2	Noisy alignments with insertion
**JAFFA - Hybrid**	LINC01420-FAAH2	No spanning reads in alignment to fusion construct

**JAFFA - Hybrid**	JPX-KTN1	Low complexity in KTN1 region (KTN1 gene has T and A repeats)
**JAFFA - Hybrid**	JPX-XIST	Low complexity (XIST part has a lot of T repeats)
**JAFFA - Hybrid**	UBE2J2-EEF1D	Homology (EEF1D part is 100% identical to other regions in transcriptome)
**JAFFA - Hybrid**	ZNF813-NTRK1	Noisy alignments
**JAFFA - Hybrid**	RSRC1-EGFR	Low complexity (The RSRC1 has a lot of AG repeats)
**JAFFA - Hybrid**	QKI-PACRG	Low complexity (PACRG part is highly repetitive)
**JAFFA - Hybrid**	RPS18-PCDHGA1	Low complexity (RPS18 part is highly repetitive)
**JAFFA - Hybrid**	EGFR-PARD3	Low frequency (0.00006%)
**JAFFA - Hybrid**	EGFR-MT-RNR2	No spanning reads in alignment to fusion construct
**JAFFA - Hybrid**	GNAI1-ISPD	Low complexity and repeats in ISPD part
**JAFFA - Hybrid**	GNAI1-PTK2	Low complexity and repeats around breakpoint and GNAI1-PTK2 part
**JAFFA - Hybrid**	UBE2J2-USP4	No spanning reads in alignment to fusion construct

**JAFFA-Hybrid**	THBS1-PCDHGA1	Low complexity (THBS1 is highly repetitive)
**JAFFA-Hybrid**	PRKCA-RNA5-8S5	No spanning reads in alignment to fusion construct
**JAFFA-Hybrid**	RAF1-ACTG1	No spanning reads in alignment to fusion construct
**JAFFA-Hybrid**	SMIM4-PCDHGA1	Low frequency (0.005%)
**JAFFA-Hybrid**	PCDHGA1-LILRB4	No spanning reads in alignment to fusion construct
**JAFFA-Hybrid**	MYB-PARG	Noisy alignments
**JAFFA-Hybrid**	QKI-MT-RNR2	Noisy alignments
**JAFFA-Hybrid**	QKI-SOX10	Noisy alignment with insertion
**JAFFA-Hybrid**	QKI-RBM4	Low complexity (Poly A repeats)
**JAFFA-Hybrid**	QKI-ZNF595	Homology (The ZNF595 part is highly identical to other regions in transcriptome)
**JAFFA-Hybrid**	QKI-SCAF8	No spanning reads in alignment to fusion construct
**JAFFA-Hybrid**	PTPRZ1-SAMD4A	Homology (The SAMD4A part is identical to other regions in transcriptome)

**JAFFA - Hybrid**	PTPRZ1-PSMB1	Low complexity (T repeats in the PSMB1 region)
**JAFFA - Hybrid**	PTPRZ1-TCAF1	Low frequency (0.00025%)
**JAFFA - Hybrid**	BRAF-MT-RNR2	No spanning reads in alignment to fusion construct
**JAFFA - Hybrid**	FGFR1-MT-RNR2	No spanning reads in alignment to fusion construct
**JAFFA - Hybrid**	MT-RNR2-NTRK2	No spanning reads in alignment to fusion construct
**JAFFA - Hybrid**	MT-RNR2-QKI	Noisy alignments
**JAFFA - Hybrid**	JPX-AC026150.8	Homology (AC026150.8 is highly homologous to other regions in transcriptome)
**JAFFA - Hybrid**	TPM3:MRPS21	Homology (TPM3 and MRPS21 part maps to other regions in transcriptome)
**JAFFA - Hybrid**	QKI:MT-RNR2	No spanning reads in alignment to fusion construct
**JAFFA - Hybrid**	QKI:LARP4B	Homology (The LARP4B part maps to other regions in transcriptome)
**JAFFA - Hybrid**	PTPRZ1:ZNF81	Homology (The ZNF81 part also maps to other regions non specific mappings)
**JAFFA - Hybrid**	PTPRZ1:RNY4	Homology (Mapping in PTPRZ1 and RNY4 multiple map to several regions across transcriptome)

**JAFFA - Hybrid**	PTPRZ1:PCDHGA8	Homology (The PTPRZ1 and PCDHGA regions multiply map to several regions across transcriptome)
**JAFFA - Hybrid**	ETV6:METTL25	Homology (The ETV6 first few bases are homologous to other regions in transcriptome)
**JAFFA - Hybrid**	PTPRZ1:RNY1	Homology (Mapping in PTPRZ1 and RNY1 multiple map to several regions across transcriptome)
**JAFFA - Hybrid**	HNRNPK:NTRK2	Homology (HNRNPK part is highly homologous to other regions of transcriptome)
**JAFFA - Hybrid**	QKI:APP	Homology (RAB2A part maps multiple to other regions)
**JAFFA - Hybrid**	RAB2A:QKI	Homology (Part of APP maps to other regions within transcriptome)
**JAFFA - Hybrid**	EGFR:STARD9	No spanning reads in alignment to fusion construct
**JAFFA - Hybrid**	EGFR:FO082796.1	Homology (FO082796.1 part maps multiply to several regions)
**JAFFA - Hybrid**	IGF2:RELA	Homology (The read multiply maps to other regions and shows up as noisy alignment)
**JAFFA - Hybrid**	RELA:IGF2	Homology (The read multiply maps to other regions and shows up as noisy alignment)
**JAFFA - Hybrid**	RELA:SNORD58B	No spanning reads in alignment to fusion construct
**JAFFA - Hybrid**	MACF1:MT-ND4	No spanning reads in alignment to fusion construct

**JAFFA - Hybrid**	QKI:MT-RNR2	Homology (The MT-RNR2 maps multiply to several regions)
**JAFFA - Hybrid**	HLA-C:HLA-A	Homology (HLA genes)
**JAFFA - Hybrid**	BRAF:MT-ND2	No spanning reads in alignment to fusion construct
**JAFFA - Hybrid**	FGFR1:RNA5-8S5	No spanning reads in alignment to fusion construct
**JAFFA - Hybrid**	MMP16:MT-CO1	No spanning reads in alignment to fusion construct
**JAFFA - Hybrid**	NTRK2:RNY1	No spanning reads in alignment to fusion construct
**JAFFA - Hybrid**	JPX:STAG2	Homology and Low complexity (STAG2 part)
**JAFFA - Hybrid**	SLC7A11-AS1:QKI	Homology (SLC7A11 part maps multiply to other regions)
**JAFFA - Hybrid**	PTPRZ1:Y_RNA	Homology (The Y_RNA part maps multiply to other regions)
**SEEKFUSION**	QKI-CASK	Homology (QKI and CASK parts map multiply to other regions)
**SEEKFUSION**	QKI:APP	Homology (RAB2A part maps multiple to other regions)
**SEEKFUSION**	RAB2A:QKI	Homology (Part of APP maps to other regions within transcriptome)

1.6 Supplementary Table 6: Gene Fusions validated

Assay Type	Expected	Observed	Assay Type	Expected	Observed
Neurological cancer	*KIAA1549-BRAF*	*KIAA1549-BRAF* E16-E9	Sarcoma	PRR12-FOXO1	PRR12-FOXO1 (11-2)
Neurological cancer	*FGFR3-TACC3*	*FGFR3-TACC3* E17-E8	Sarcoma	NAB2-STAT6	NAB2-STAT6 (4-2)
Neurological cancer	*EGFR-EGFR* E1-E8	*EGFR-EGFR* E1-E8	Sarcoma	NAB2-STAT6	NAB2-STAT6 (4-2)
Neurological cancer		*EGFR-PSPH* E24-E8	Sarcoma	NAB2-STAT6	NAB2-STAT6 (4-2)
Neurological cancer		*EGFR-FKBP9L* E7-E4	Sarcoma	NAB2-STAT6	NAB2-STAT6 (6-17)
Neurological cancer		*EGFR-SEPT14* E24-E10	Sarcoma	NAB2-STAT6	NAB2-STAT6 (6-17)
Neurological cancer	*KIAA1549-BRAF*	*KIAA1549-BRAF* E15-E9	Sarcoma	NAB2-STAT5	NAB2-STAT5 (6-16)
Neurological cancer	*KIAA1549-BRAF*	*KIAA1549-BRAF* E16-E11	Sarcoma		
Neurological cancer	*SRGAP3-RAF1*	*SRGAP3-RAF1* E10-E9	Sarcoma	NAB2-STAT6	NAB2-STAT6 (6-16)
Neurological cancer	*EGFR-SEPT14*	*EGFR-SEPT14* E24-E10	Sarcoma	SRSF3-USP6	SRSF3-USP6 (1-10)
Neurological cancer	*KIAA1549-BRAF*	*KIAA1549-BRAF* E16-E9	Sarcoma	CDH11-USP6	CDH11-USP6 (1-9)
Neurological cancer	*KIAA1549-BRAF*	*KIAA1549-BRAF* E15-E9	Sarcoma	EWSR1-CREB1	EWSR1-CREB1 (7-6)
Neurological cancer	*EGFR-EGFR* E1-E8	*EGFR-EGFR* E1-E8	Sarcoma	ASPSCR1-TFE3	ASPSCR1-TFE3 (7-5)
Neurological cancer	*KIF21B-NTRK1*	*KIF21B-NTRK1* E14-E10	Sarcoma	EWSR1-ATF1	EWSR1-ATF1 (7-5)
Neurological cancer	*YAP1* rearrangement	*YAP1-FAM118B* E7-E3	Sarcoma		
Neurological cancer	*EGFR-EGFR* E1-E8	*EGFR-EGFR* E1-E8	Sarcoma	EWSR1-ATF1	EWSR1-ATF1 (11-3)
Neurological cancer	*C11orf95-RELA*	*C11orf95-RELA* E3-E3	Sarcoma	WWTR1-CAMTA1	WWTR1-CAMTA1 (3-8)
Neurological cancer	*C11orf95-RELA*	*C11orf95-RELA* E3-E3	Sarcoma	WWTR1-CAMTA1	WWTR1-CAMTA1 (3-8)
Neurological cancer	*FGFR1-TACC1*	*FGFR1-TACC1* E18-E7	Sarcoma	ETV6-NTRK3	ETV6-NTRK3 (5-15)
Neurological cancer	*KIAA1549-BRAF*	*KIAA1549-BRAF* E16-E9	Sarcoma	ETV6-NTRK3	ETV6-NTRK3 (5-15)

Neurological cancer	*PDE4B-NTRK2*	*PDE4B-NTRK1* E9-E15	Sarcoma	COL1A1-PDGFB	COL1A1-PDGFB (25-2)
Neurological cancer	*KIAA1549-BRAF*	*KIAA1549-BRAF* E15-E11	Sarcoma	COL1A1-PDGFB	COL1A1-PDGFB (46-2)
Neurological cancer	*KIAA1549-BRAF*	*KIAA1549-BRAF* E16-E9	Sarcoma	CIC-DUX4	CIC-DUX4 (21-1)
Neurological cancer	*QKI-RAF1*	*QKI-RAF1* E3-E8	Sarcoma	CIC-DUX4	CIC-DUX4 (21-1)
Neurological cancer	*FGFR3-TACC3*	*FGFR3-TACC3* E17-E10	Sarcoma	ZC3H7B-BCOR	ZC3H7B-BCOR (10-7)
Neurological cancer	*EGFR-EGFR* E1-E8	*EGFR-EGFR* E1-E8	Sarcoma		
Neurological cancer	*FGFR1-TACC1*	*FGFR1-TACC1* E18-E7	Sarcoma	ZC3H7B-BCOR	ZC3H7B-BCOR (10-7)
Neurological cancer	*FGFR3-TACC3*	*FGFR3-TACC3* E17-E11	Sarcoma	EWSR1-NR4A3	EWSR1-NR4A3 (12-3)
Neurological cancer	*EGFR-EGFR* E1-E8	*EGFR-EGFR* E1-E8	Sarcoma	EWSR1-NR4A3	EWSR1-NR4A3 (12-3)
Neurological cancer	*QKI-NTRK2*	*QKI-NTRK2* E6-E15	Sarcoma	FUS-CREB3L1	FUS-CREB3L1 (6-5)
Neurological cancer	*KIAA1549-BRAF*	*KIAA1549-BRAF* E16-E9	Sarcoma	FUS-CREB3L2	FUS-CREB3L2 (7-5)
Neurological cancer	*NAB2-STAT6*	*NAB2-STAT6* E4-E2	Sarcoma	FUS-CREB3L2	FUS-CREB3L2 (6-5)
Neurological cancer	*NAB2-STAT6*	*NAB2-STAT6* E4-E2	Sarcoma	PAX3-FOXO1	PAX3-FOXO1 (7-2)
Neurological cancer	*NAB2-STAT6*	*NAB2-STAT6* E4-E2	Sarcoma	PAX3-NCOA1	PAX3-NCOA1 (6-13)
Neurological cancer	*EWSR1-FLI1* E7-E6	*EWSR1-FLI1* E7-E6	Sarcoma	PAX3-MAML3	PAX3-MAML3 (7-2)
Neurological cancer	*EWSR1-FLI1* E7-E6	*EWSR1-FLI1* E7-E6	Sarcoma	PAX3-NCOA2	PAX3-NCOA2 (7-12)
Neurological cancer	*EWSR1-FLI1*	*EWSR1-FLI1* E7-E6	Sarcoma		
Neurological cancer	*SRGAP3-RAF1* E10-E9	*SRGAP3-RAF1* E10-E9	Sarcoma	FUS-DDIT3	FUS-DDIT3 (7-2)
Neurological cancer	*KIAA1549-BRAF* E16-E9	*KIAA1549-BRAF* E16-E9	Sarcoma	SFPQ-FOSB	SFPQ-FOSB (9-2)
Neurological cancer	*MYB-QKI* E10-E5	*MYB-QKI* E10-E5	Sarcoma	FUS-DDIT3	FUS-DDIT3 (5-2)

Neurological cancer	*FAM131B-BRAF* E2-E9	*FAM131B-BRAF* E2-E9	Sarcoma	EP400-PHF1	EP400-PHF1 (37-2) EP400-PHF1 (37-2)
Neurological cancer	*PVT1-MYC* E3-E2	*PVT1-MYC* E4-E2	Sarcoma	JAZF1-SUZ12	JAZF1-SUZ12 (3-2)
Neurological cancer	*FGFR1-TACC1* E18-E7	*FGFR1-TACC1* E18-E7	Sarcoma	JAZF1-PHF1	JAZF1-PHF1 (3-2)
Neurological cancer	*DDX31-GFI1B* E19-E2	*DDX31-GFI1B* E19-E2	Sarcoma		
Neurological cancer	*SLC44A1-PRKCA* E15-E9	*SLC44A1-PRKCA* E15-E9	Sarcoma	RANBP2-ALK	RANBP2-ALK (18-20)
Neurological cancer	*MYB-ESR1* E15-E6	*MYB-ESR1* E14-NA	Sarcoma	TRAF3-ALK	TRAF3-ALK (11-20)
Neurological cancer	*PTPRZ1-MET* E1-E2	*PTPRZ1-MET* E1-E2	Sarcoma	IGFBP5-ALK	IGFBP5-ALK (1-19)
Neurological cancer	*TPM3-NTRK1* E7-E10	*TPM3-NTRK1* E7-E10	Sarcoma	MPRIP-ALK	MPRIP-ALK (21-20)
Neurological cancer	*MYB-PCDHGA1* E9-E2	*MYB-PCDHGA1* E9-E2	Sarcoma	AHRR-NCOA2	AHRR-NCOA2 (9-11)
Neurological cancer	*FGFR1-FGFR1* E9-E19	*FGFR1-FGFR1* E9-E19*	Sarcoma	AHRR-NCOA2	AHRR-NCOA2 (10-13)
Sarcoma	MIR143HG-NOTCH2	MIR143HG-NOTCH2 (1-	Sarcoma	EWSR1-ETV1	EWSR1-ETV1 (7-10)
Sarcoma			Sarcoma	EWSR1-CREB3L1	EWSR1-CREB3L1 (8-6)
Sarcoma	GLI1-ACTB	GLI1-ACTB (6-3)	Sarcoma	FUS-CREB3L1	FUS-CREB3L1 (int 6-6)
Sarcoma	HMGA2-LPP	HMGA2-LPP (3-9)			

1.7 Supplementary Table 7: Fusions reported in sarcoma and neurological cancer clinical cases

EGFR VIII	WWTR1-CAMTA1	BCR-NTRK2	EIF1-USP6
KIAA1549-BRAF	PAX3-MAML3	ARHGEF2-NTRK1	FUS-ERG
FGFR3-TACC3	FUS-CREB3L2	MN1-MOB3B	RAB2A-BCS1L
PTPRZ1-MET	ACTB-GLI1	MDM4-GLI1	PRRC2B-ALK
C11orf95-RELA	EP400-PHF1	EML4-NTRK3	CIC-FOXO4
FGFR1-TACC1	ZC3H7B-BCOR	CAND1-EGFR	CALD1-USP6
EGFR-SEPT14	PHF1-TFE3	TRIM24-MET	ATIC-ALK
CAPZA2-MET	MYH9-USP6	PTTG1IP-BRAF	GLI1-ACTB
NAB2-STAT6	EML4-ALK	FGFR3-FAM184B	HMGA2-RAB3IP
GTF2I-BRAF	EWSR1-CREB3L1	FGFR3-PLEC	EWSR1-BEND2
ATG7-RAF1	EWSR1-NR4A3	TPR-NTRK1	CIC-DUX4L4
GKAP1-NTRK2	EWSR1-PBX3	MBNL1-RAF1	CNBP-PDCD11

SLC44A1-PRKCA	PAX7-FOXO1	EVI5-BRAF	MYBL1-NFIB
AFAP1-NTRK2	TAF15-NR4A3	PID1-BRAF	TFG-NTRK3
GNAI1-BRAF	RNF213-ALK	FGFR1-FGFR1	TFG-ZBTB10
ZSCAN21-MET	MXD4-NUTM1	EWSR1-FLI1	YAP1-C15orf55
EWSR1-CREB1	MIR143HG-NOTCH2	EWSR1-ERG	NCOA2-SPIDR
TRIO-NTRK2	JAZF1-SUZ12	SS18-SSX1	SRF-NCOA2
LACE1-QKI	ETV6-NTRK3	EWSR1-WT1	EWSR1-GFI1B
EWSR1-ETV1	BCOR-ZC3H7B	PAX3-FOXO1	EWSR1-CREB3L2
MN1-BEND2	AHRR-NCOA2	EWSR1-ATF1	HMGA2-LEMD3
YAP1-MAML2	YWHAE-NUTM2B	COL1A1-PDGFB	EWSR1-POU5F1P3
ST7-MET	EWSR1-NFATC2	FUS-DDIT3	RBM10-TFE3
BCAN-NTRK1	EWSR1-TFCP2	BCOR-CCNB3	FBXO32-PLAG1
PRKAR2B-BRAF	LMNA-NTRK1	HEY1-NCOA2	AHRR-EXOC3
MYB-QKI	MEAF6-PHF1	SRGAP3-RAF1	MEIS1-NCOA2
YAP1-FAM118B	CIC-DBET	KCTD16-NTRK2	FUS-NFATC2
EWSR1-CREM	EWSR1-PATZ1	MEF2D-NTRK1	EPC1-PHF1
VCAN-NTRK2	EWSR1-FEV	HEPACAM2-MET	MIR22HG-USP6
LNX1-PDGFRA	WWTR1-FOSB	TMEM165-PDGFRA	TPM4-ALK
MYB-PCDHGA1	MIR143HG-MYC	EGFR-ELDR	C11orf95-MKL2
PTPN12-MET	CIC-MIR8078	ILF3-ELAVL3	PAX3-NCOA1
QKI-RAF1	EWSR1-COLCA2	GALNT13-PRKCA	ZFP64-NCOA2
TPM3-NTRK1	JAZF1-PHF1	DCTN2-GLI1	PGR-NR4A3
MTSS1-BRAF	EWSR1-POU5F1	EGFR-GRIP1	MBTD1-CXorf67
BRAF-GTF2IP1	YAP1-TFE3	ATG7-UBXN7	EWSR1-FOXJ3
PTPRZ1-LINC00278	NOTCH2NL-NOTCH2	FGFR3-CGNL1	CHCHD7-PLAG1
AKAP9-BRAF	FUBP1-TFE3	C8orf34-SULF1	PAX3-NCOA2
PTPRZ1-CUL1	LRRFIP1-ALK	SCAPER-BRAF	PRRX1-NCOA1
CUX1-MET	FUS-TFCP2	FGFR3-BRAP	CIC-DUX2
FRS2-GLI1	FN1-FGFR1	FGFR1-ASPA	COL1A1-SSH3
FGFR3-LRRC48	SPARC-USP6	STRN3-NTRK2	PRRX1-NCOA2
MN1-CXXC5	FUS-ETV1	CLIP2-MET	ST3GAL2-FOXO1
QKI-AGPAT4	ASPSCR1-TFE3	FAM131B-BRAF	DYNC1I2-ALK
SMURF1-MET	BCOR-ITD	EGFR-DPY19L1P1	EWSR1-DDIT3
SEC61G-EGFR	BRD4-C15orf55	MTM1-MAMLD1	SNAI2-PLAG1
DPP6-MET	TFG-ROS1	SLC44A1-BRAF	TRPS1-PLAG1
AGK-BRAF	EWSR1-PBX1	NPM1-BRAF	PUF60-TFE3
SOX6-RAF1	SQSTM1-ALK	NRCAM-MET	ACCN2-ATF1
PDGFRA-CHIC2	RANBP2-ALK	MKRN1-BRAF	ESR1-NR4A3
QKI-PACRG	HMGA2-SNRPF	RIN2-BRAF	DCTN1-ALK
MYBL1-MAML2	EGFL7-FOSB		

2 Supplementary methods

2.1 Specimen requirements:

This assay requires at least 10% tumor nuclei. Preferred amount of tumor area with sufficient percent tumor nuclei: tissue 144 mm(2). Minimum amount of tumor area: tissue 36 mm(2). These amounts are cumulative over up to 10 unstained slides and must have adequate percent tumor nuclei.Tissue fixation: 10% neutral buffered formalin, not decalcified

2.2 Preferred Specimen Type:

Specimen Type: Formalin-fixed, paraffin-embedded (FFPE) tissue. Container/Tube: Tissue block. Collection Instructions: Submit a formalin-fixed, paraffin-embedded tissue block.

2.3 Acceptable Specimen Type:

Specimen Type: FFPE Tissue. Slides: 1 Stained and 10 unstained. Collection Instructions: Submit 1 slide stained with hematoxylin and eosin and 10 unstained, nonbaked slides with 5-micron thick sections of the tumor tissue. Specimen Type: Cytology slide (direct smears or ThinPrep). Slide: 1 to 3 slides. Collection Instructions: Submit 1 to 3 slides stained and coverslipped with a preferred total of 5000 nucleated cells or a minimum of at least 3000 nucleated cells. Note: Glass coverslips are preferred; plastic coverslips are acceptable but will result in longer turnaround times.

2.4 Deduping methods:

The UMI consensus is built as follows:

Say, we denote the real (but unknown) base as $R \in S$ where $S = [A, T, G, C]$ is our alphabet. Denote the observed bases $O_n \in S, n = 1, \ldots, N$and their corresponding quality scores$Q_n \in R_+ \; n = 1, \ldots, N$. The quality scores can be converted into their corresponding error probabilities $P_n^{err} = 10^{\frac{-Q_n}{10}}, n = 1, \ldots N$. Then we use Bayes' theorem to iteratively update our knowledge about the real base R. Specifically, we assign a prior distribution $P[R = A] = P[R = T] = \frac{1-P^{GC}}{2}$ and $P[R = G] = P[R = C] = P^{GC}/2$, where the GC percent of the region P^{GC} can be computed genome-wide. Then for each observation we apply Bayes' theorem to update our knowledge, starting with the first observation:

$$P[R = r|O_1 = o_1] = \frac{P[O_1 = o_1|R = r]P[R=r]}{\sum_{x \in S} P[O_1 = o_1|R = x]P[R=x]}, r \in S, o1 \in S$$

Where the likelihood probabilities can be computed from P_1^{err} as

$$P[O_1 = o_1|R = r] = [1 - P_1^{err}]\delta_{o_1,r} + \frac{P_1^{err}[1 - \delta_{o_1,r}]}{3}, r \in S, o_1 \in S$$

Where $\delta_{o_1,r}$ is the Kronicker delta that equals one when its subscripts are equal and zero otherwise. Then at stage n where $P[r|o_1, \ldots., o_n]$ is computed, the next observation o_{n+1} for each $r \in S$ using assumption that observations of sequences are independent of each other.

$$P[r|o_1 \ldots, o_{n+1}] = \frac{(P[o_{n+1}\ |r, o_1 \ldots, o_n]P[r|o_1 \ldots, o_n])}{\sum_{x \in S}(P[o_{n+1}\ |x, o_1 \ldots, o_n]P[x|o_1 \ldots, o_n])}$$

$$= \frac{P[o_{n+1}|r]P[r|o_1 \ldots, o_n]}{\sum_{x \in S}(P[o_{n+1}\ |x]P[x|o_1 \ldots, o_n])}$$

Finally, once we have iterated through all our observations, we make a consensus estimate for the real base $\hat{R}$ using the maximum a posteriori (MAP) estimate: $\hat{R} = argmax_{R \in S} P[r|o_1 \ldots o_n]$ and the corresponding updated error probability estimate is given by: $\widehat{P^{err}} = 1 - P[\hat{R}|o_1 \ldots o_n]$ which provides updated score estimate: $\widehat{Q_n} = -10 log_{10} (\widehat{P^{err}})$.

Mononucleotide repeat ratio calculation:

Say there is a sequence where there are 3 mononucleotides that are repeated

$(X_i, X_{i+1}, X_{i+2}, X_{i+3}, X_{i+4}, X_{i+5} \ldots X_n)$ where $X_i, \cdots, X_n \epsilon\ [A, T, G, C]$ and $X_{i+2} = X_{i+3} = X_{i+4}$ $are\ the\ repeated\ mononucleotides$

Each nucleotide is assigned a score, $S = 1$ and for the above sequence the mononucleotide repeat ratio is defined by the formula:

$$MRR = \frac{\{S_i^2 + S_{i+1}^2 + (S_{i+2} + \ S_{i+3} + \ S_{i+4})^2 + \ S_{i+5}^2 + \cdots + S_n^2\}}{n}$$

For a region with no mononucleotide repeats, the MRR value is equal to 1. For a region with high repeats the value increases based on the length of mononucleotide repeat signatures. For every assembled contig that mapped to a region in the genome, the region is assessed for mononucleotide repeat ratio and the regions with ratio greater than an empirically chosen threshold of 10 are not considered as potential fusion partners.

2.5 UMI and Adapter sequence trimming

UMIs are 1st 12 bases of read 2 in the chemistry.

```
>R1_adapter
AGGACTCCAAT
>R2_adaper
CAAAACGCAATACTGTACATT
>R2_adapter_rev
AATGTACAGTATTGCGTTTTG
>polyA
AAAAAAAAAAAAAAAAAAAAAAAAAAAAAAAAAAAAAAAAAAAAAAA
>polyT
TTTTTTTTTTTTTTTTTTTTTTTTTTTTTTTTTTTTTTTTTTTTTTT
```

The adapter and UMIs were trimmed using FASTP.

3 Raw data availability

All benchmarking raw data has been uploaded to NCBI's SRA website.

NCBI SRA ID	URL	SampleID in study
21386667	https//www.ncbi.nlm.nih.gov/sra/21386667	pos_S1_KIAA1549fusionBRAF
21386668	https//www.ncbi.nlm.nih.gov/sra/21386668	pos_S11_QKIfusionRAF1
21386669	https//www.ncbi.nlm.nih.gov/sra/21386669	pos_S10_PDE4BfusionNTRK2
21386670	https//www.ncbi.nlm.nih.gov/sra/21386670	pos_S12_NAB2fusionSTAT6

21386671	https//www.ncbi.nlm.nih.gov/sra/21386671	pos_S7_MN1fusionMOB3B
21386672	https//www.ncbi.nlm.nih.gov/sra/21386672	pos_S6_FGFR3fusionTACC3
21386673	https//www.ncbi.nlm.nih.gov/sra/21386673	pos_S4_EWSR1fusionFLI1
21386674	https//www.ncbi.nlm.nih.gov/sra/21386674	pos_S2_EGFRfusionSEPT14
21386675	https//www.ncbi.nlm.nih.gov/sra/21386675	pos_S3_EGFR1fusionEGFR8
21386676	https//www.ncbi.nlm.nih.gov/sra/21386676	pos_S9_C11ORF95fusionRELA
21386677	https//www.ncbi.nlm.nih.gov/sra/21386677	pos_S5_BRAFfusionFAM131B
21386678	https//www.ncbi.nlm.nih.gov/sra/21386678	pos_S13_POSITIVEfusionCONTROL
21386679	https//www.ncbi.nlm.nih.gov/sra/21386679	pos_S8_SRGAP3fusionRAF1
21386680	https//www.ncbi.nlm.nih.gov/sra/21386680	pos_S14_Insilico
21386681	https//www.ncbi.nlm.nih.gov/sra/21386681	neg_S1_sample1
21386682	https//www.ncbi.nlm.nih.gov/sra/21386682	neg_S2_sample2
21386683	https//www.ncbi.nlm.nih.gov/sra/21386683	neg_S3_sample3
21386684	https//www.ncbi.nlm.nih.gov/sra/21386684	neg_S4_sample4
21386685	https//www.ncbi.nlm.nih.gov/sra/21386685	neg_S5_sample5
21386686	https//www.ncbi.nlm.nih.gov/sra/21386686	neg_S6_sample6
21386687	https//www.ncbi.nlm.nih.gov/sra/21386687	neg_S7_sample7
21386688	https//www.ncbi.nlm.nih.gov/sra/21386688	neg_S8_sample8
21386689	https//www.ncbi.nlm.nih.gov/sra/21386689	neg_S9_sample9

4 Docker Accessiblity and instructions to run pipeline in docker

4.1 Instructions to use it in linux machine/mac/windows:

- Create a new account if you don't have one here - https://hub.docker.com/signup
- Download and install docker as per operating system use
- Make sure that in the docker config the memory is set to at least 6GB
- Open terminal, and pull from seekfusion docker–
    - docker pull jagadhesh89/seekfusion
- Ensure that you are running jagadhesh89/seekfusion
    - docker run -i -t jagadhesh89/seekfusion
- Run the pipeline with files in the docker image (this command also available on dockerhub page)
    - pipeline/v2.00.00/src/NGS_UMIFUSION/main/shell/runFusion.sh -i /pipeline/test_data/ -m local
    - The outputs are available in /pipeline/test_data under umifusion directory.
    - The vcf file output is under under /pipeline/test_data/umifusion/reports/*.vcf
    - The sample data contains the EWSR1-NR4A3 fusion and NTRK2-VCL fusion.
    - The sample output directory needs to be backed up, renamed or removed while testing.

4.2 Screenshots

```
(base) R5192924:Data m139105$ docker run -i -t jagadhesh89/seekfusion
[root@df813058159f /]# rm -rf /pipeline/test_data/umifusion/
[root@df813058159f /]# /pipeline/v2.00.00/src/NGS_UMIFUSION/main/shell/runFusion.sh -i /pipeline/test_data/ -m local
Starting runFusion.sh

 Pipeline preprocessing
Using configuration file at: /pipeline/v2.00.00/src/umifusion.profile
Using common functions: /pipeline/v2.00.00/src/NGS_PIPELINE_UTILITIES/src/main/bash/commonFunctions.sh
[2021-09-16T18:25:58+0000] [INFO] [runFusion.sh]        Ordered service folder is in the sample directory and used as configuration
[2021-09-16T18:25:58+0000] [INFO] [runFusion.sh]        Command run: runFusion.sh -i /pipeline/test_data/ -m local
[2021-09-16T18:25:58+0000] [INFO] [runFusion.sh]         Pre execution validation
[2021-09-16T18:25:58+0000] [INFO] [runFusion.sh]        fastqUmiDedup option in os.cfg is \'yes\' - calling Fastq UMI Dedup task.
[2021-09-16T18:25:58+0000] [INFO] [runFusion.sh]        Pipeline Run Details

SAMPLE_NAME:            test_data

SCRIPT DATA

SCRIPT_HOME:            /pipeline/v2.00.00/src/NGS_UMIFUSION/main
SOURCE_HOME:            /pipeline/v2.00.00/src
TOOLS_HOME:             /pipeline/v2.00.00/tools
CONFIGS_HOME:           /pipeline/v2.00.00/configs
REFERENCES:             /reference/snapshot_v2
TEMP_LOG_SPACE:         /logs/umifusion
RESOURCES_HOME:         /pipeline/v2.00.00/src/NGS_UMIFUSION/main/resources

SGE DATA

QSUB_QUEUE:             prod.q
QSUB_TIMEOUT:           86400
CROMWELL S_RT:          47:55:00
CROMWELL H_RT:          48:00:00
CROMWELL QSUB OPTIONS:  -l umi_fusion=1
```

```
ORDERED SERVICE DATA

TEST_DEF_HOME:          /testDefinition
RECIPE:                 TEST
MASTER_PANEL:
PROJECT_NAME:           TESTRUN
OS_CONFIG:              /pipeline/test_data/umifusion/configs/os.cfg
OS_PIPELINE_DIR:        /pipeline/test_data/ordered_service/pipelines/umifusion

WDL DATA

JSON_INPUT:             /pipeline/test_data/umifusion/configs/inputs.json
JSON_CONFIG:            /pipeline/test_data/umifusion/configs/configs.json
CROMWELL_FOLDER:        /temp/cromwell
CROMWELL_LOGS:          /logs/umifusion
SGE_CONFIG:             /pipeline/v2.00.00/configs/local.conf
OUTPUT_JSON:            /pipeline/test_data/umifusion/configs/outputs.json

RESULTS

OUTPUT_DIR:             /pipeline/test_data/umifusion
CONFIG_DIR:             /pipeline/test_data/umifusion/configs
DEVICE_NAME:            M04551


[2021-09-16T18:25:58+0000] [INFO] [runFusion.sh]         Beginning processing
[2021-09-16T18:25:58+0000] [INFO] [runFusion.sh]        Running Command:

[2021-09-16T18:25:58+0000] [INFO] [runFusion.sh]        /usr/bin/java     -Dbackend.providers.SGE.config.root=/temp/cromwell     -Dworkflow-options.workflow-
log-dir=/logs/umifusion     -Dconfig.file=/pipeline/v2.00.00/configs/local.conf     -Xmx8G     -Xms4G     -Xss4M     -jar /biotools/cromwell/cromwell-49.jar
    run /pipeline/v2.00.00/src/NGS_UMIFUSION/main/wdl/fusion.wdl     -p /pipeline/v2.00.00/src/NGS_UMIFUSION/src.zip     -i /pipeline/test_data/umifusion/con
figs/inputs.json     -o /pipeline/test_data/umifusion/configs/configs.json     -m /pipeline/test_data/umifusion/configs/outputs.json | tee -a /pipeline/test_
data/umifusion/logs/main.log
```
